# Managing Unusual Sensory Experiences in People with First-Episode Psychosis (MUSE FEP): a study protocol for a single-blind parallel-group randomised controlled feasibility trial

**DOI:** 10.1136/bmjopen-2022-061827

**Published:** 2022-05-16

**Authors:** Robert Dudley, Guy Dodgson, Stephanie Common, Lucy O’Grady, Florence Watson, Christopher Gibbs, Bronia Arnott, Charles Fernyhough, Ben Alderson-Day, Emmanuel Ogundimu, Ehsan Kharatikoopaei, Victoria Patton, Charlotte Aynsworth

**Affiliations:** 1 Newcastle University Population Health Sciences Institute, Newcastle upon Tyne, UK; 2 Early Intervention in Psychosis service, Cumbria, Northumberland, Tyne and Wear NHS Foundation Trust, Newcastle Upon Tyne, UK; 3 Early Intervention in Psychosis, Tees Esk and Wear Valley NHS Foundation Trust, Darlington, UK; 4 Research and Development, Tees Esk and Wear Valley NHS Foundation Trust, Darlington, UK; 5 Department of Psychology, University of Durham, Durham, UK; 6 Department of Mathematical Sciences, Univeristy of Durham, Durham, UK

**Keywords:** Schizophrenia & psychotic disorders, Adult psychiatry, Clinical trials

## Abstract

**Introduction:**

Hallucinations (hearing or seeing things that others do not) are a common feature of psychosis, causing significant distress and disability. Existing treatments such as cognitive–behavioural therapy for psychosis (CBTp) have modest benefits, and there is a lack of CBTp-trained staff. Shorter, targeted treatments that focus on specific symptoms delivered by a non-specialist workforce could substantially increase access to treatment.

Managing Unusual Sensory Experiences (MUSE) explains why people have hallucinations and helps the person to develop and use coping strategies to reduce distress. MUSE focuses only on hallucinations, and treatment is short (four to six, 1-hour sessions per week). It is a digital intervention, run on National Health Service (NHS) laptops, which provides information about hallucinations in an engaging way, using audio, video and animated content. Crucially, it is designed for use by non-specialist staff like community psychiatric nurses.

**Methods and analysis:**

The study is a two-arm feasibility randomised controlled trial comparing MUSE and treatment as usual (TAU) (n=40) to TAU alone (n=40), recruiting across two NHS Trusts, using 1:1 allocation and blind assessments before and after treatment (2 months) and at follow-up (3 months). Quantitative information on recruitment rates, adherence and completion of outcome assessments will be collected. Qualitative interviews will capture service users’ experience of therapy and clinicians’ experiences of the training and supervision in MUSE. Clinicians will also be asked about factors affecting uptake, adherence and facilitators/barriers to implementation. Analyses will focus on feasibility outcomes and provide initial estimates of intervention effects. Thematic analysis of the qualitative interviews will assess the acceptability of the training, intervention and trial procedures.

**Ethics and dissemination:**

The trial has received NHS Ethical and Health Research Authority approval. Findings will be disseminated directly to participants and services, as well as through peer-reviewed publications and conference presentations.

**Trial registration number:**

ISRCTN16793301.

**Strengths and limitations of this study**

- The Managing Unusual Sensory Experiences in People with First-Episode Psychosis trial is the first digital treatment for distressing hallucinations delivered by front-line staff.
- Patient and public involvement has been woven in throughout the development of the treatment and in the design and running of the study.
- This randomised controlled multisite single-blind trial targets proposed causal factors implicated in the development of hallucinations.
- The follow-up period is short, meaning that we cannot determine whether any benefits endure.
- There is no active control, so we do not know what factors contribute to any change.

## Background

Almost all people with psychosis at some stage report hallucinations (hearing or seeing things that others do not.^1^ While for some these experiences can be positive,^2^ for many others these hallucinations can be distressing and disabling.^3^ To address this need, treatments like cognitive–behavioural therapy for psychosis (CBTp) are recommended.^4^ A recent meta-analysis^5^ indicated that CBTp has a specific and beneficial effect on hallucinations in comparison to active and inactive control conditions (Hedges’ *g*=0.34). While welcome, the effects are still relatively modest. Moreover, access to CBTp remains limited^6^ owing to the number of therapists available and the time it requires to deliver treatment.^7^ CBTp is meant to be offered as 16–24 sessions per week offered over 6–9 months.

Two approaches can help. The first is to provide briefer interventions, allowing more people to receive treatment. However, the benefits of CBTp are already modest and dose of therapy is associated with outcome.^8^ To retain the impact in a briefer form, it is essential that shorter treatments are sufficiently potent, which will likely involve targeting key causal mechanisms^9^ leading to hallucinations. The second approach is to increase access by enabling a larger and less well-trained workforce to provide treatment. The present study combines these approaches.

In terms of causal mechanisms, the exact reasons people see and hear things others do not are unclear. However, research on auditory hallucinations has shown that they take different forms that may respond to specific therapeutic approaches. A review by the International Consortium on Hallucinations Research^10^ outlined at least three different potential routes to experiencing voices for people with psychosis which may be suited to different kinds of tailored intervention: *inner speech* voices (postulated to result from the misattribution of ordinary inner speech to an external agent), *hypervigilance* voices (resulting from a biased attention to environmental stimuli) and *memory* voices (the result of intrusions from traumatic memory^11^).

The present approach draws on this understanding and uses it in a novel, digital toolkit called Managing Unusual Sensory Experiences (MUSE) that focuses only on hallucinations. MUSE uses psychoeducation about the currently known causal mechanisms of hallucination as means of exploring, with service users, why their specific experiences may be happening. This knowledge is then matched to specific, tailored interventions and coping strategies that enable the person to understand and manage their experiences differently and reduce their distress. This process relies on psychoeducation as its basis, but it is more fundamentally about helping a person to change their understanding, manage their experiences better and thus cope more effectively. MUSE is a departure from traditional CBTp as it focuses only on hallucinations and reduces the typical number of sessions to four to six. This helps prevent the ‘drift’ that can occur in treatment when there is not a specific target symptom or clear conceptual model underpinning treatment.

To help increase access to therapy, attempts have been made to train non-psychological therapy staff such as community psychiatric nurses (CPNs) in the delivery of talking therapies.^12^ However, such efforts have not demonstrated the anticipated benefits, owing to the challenge of managing high caseloads and of providing brief training for a complex intervention and a lack of standardisation of delivery.^13 14^ Recently, brief interventions (defined as less than 12 sessions^15^) provided by assistant psychologists for hallucinations have been trialled with some encouraging findings,^16^ indicating that brief targeted therapies can be provided by non-psychological therapists.

The present research continues to address this unmet need by putting high-quality treatment tools in the hands of care coordinators working in early intervention in psychosis (EIP) services. Care coordinators (who are often CPNs) are the most widely available workforce in EIP teams^6^ potentially providing the maximum access to high-quality care. EIP care coordinators work with smaller caseloads (12–15) than in other services, meaning there is capacity to deliver the treatment. Using the benefits of digital technology, we manualised and loaded MUSE onto an National Health Service (NHS) laptop, meaning that treatment is standardised, reducing the training required and the risk that staff will not feel confident in delivery. MUSE presents the content in a user-friendly, engaging way and is designed for joint use of therapist and service user in the therapy session. Clinicians use prepared videos and other types of media to help explain why the person may see or hear things others do not, and to help service users to practise coping strategies and carry out behavioural experiments to test the reality of their experiences.

An earlier version of MUSE was delivered to people with distressing voices by psychological therapists (not care coordinators who will deliver the toolkit in this project) in a recently completed uncontrolled single group feasibility study Managing Unusual Sensory Experiences in Psychosis (MUSE PSYCHOSIS^17^). Twelve participants who completed follow-up after 10 MUSE sessions reported high treatment satisfaction and acceptability (87%–90% mean overall ratings). The within-subject effect size for reducing auditory hallucination severity was large (d=0.7). Even greater benefits were evident on a recently completed uncontrolled study in which MUSE was used by psychological therapists with a group of people in an at-risk mental state for psychosis.^18^ The effect size was d=0.8 (n=19) for reduction of the intensity of auditory hallucinations, and satisfaction ratings were 90%. These studies provide encouraging evidence of the value of this approach when delivered by expert therapists.

Learning from these studies, we revised the toolkit in three important ways. First, MUSE now incorporates more information about a range of hallucinations and not just auditory.^19^ Second, the lived-experience input was strengthened to improve the relevance and accessibility of the MUSE materials. Third, MUSE was made easier to use for front-line staff, and specialist training and supervision for these staff were developed to enable them to use the MUSE toolkit.

The treatment now requires testing in a randomised trial to determine the effects on hallucinations and whether access can be improved if delivered by a non-specialist workforce. Consequently, the present study investigates the feasibility and acceptability of MUSE treatment delivered by CPNs in participants experiencing first-episode psychosis.

### Objectives

The primary objective of the present study was to establish if it is possible to undertake a larger, definitive trial in the future. Therefore, we will collect data to inform the feasibility and acceptability of a targeted hallucination-focussed treatment delivered by non-psychological therapists to establish the key parameters for a future definitive trial. The study will use mixed methods to (1) assess the feasibility of CPN staff training and delivery of the MUSE toolkit; (2) assess the acceptability of the MUSE toolkit to patients and staff; and (3) collect data to inform the effectiveness of future definitive trial (including referral, recruitment, uptake and attrition rates).

## Methods and analysis

### Trial design and flowchart

**Figure 1:**
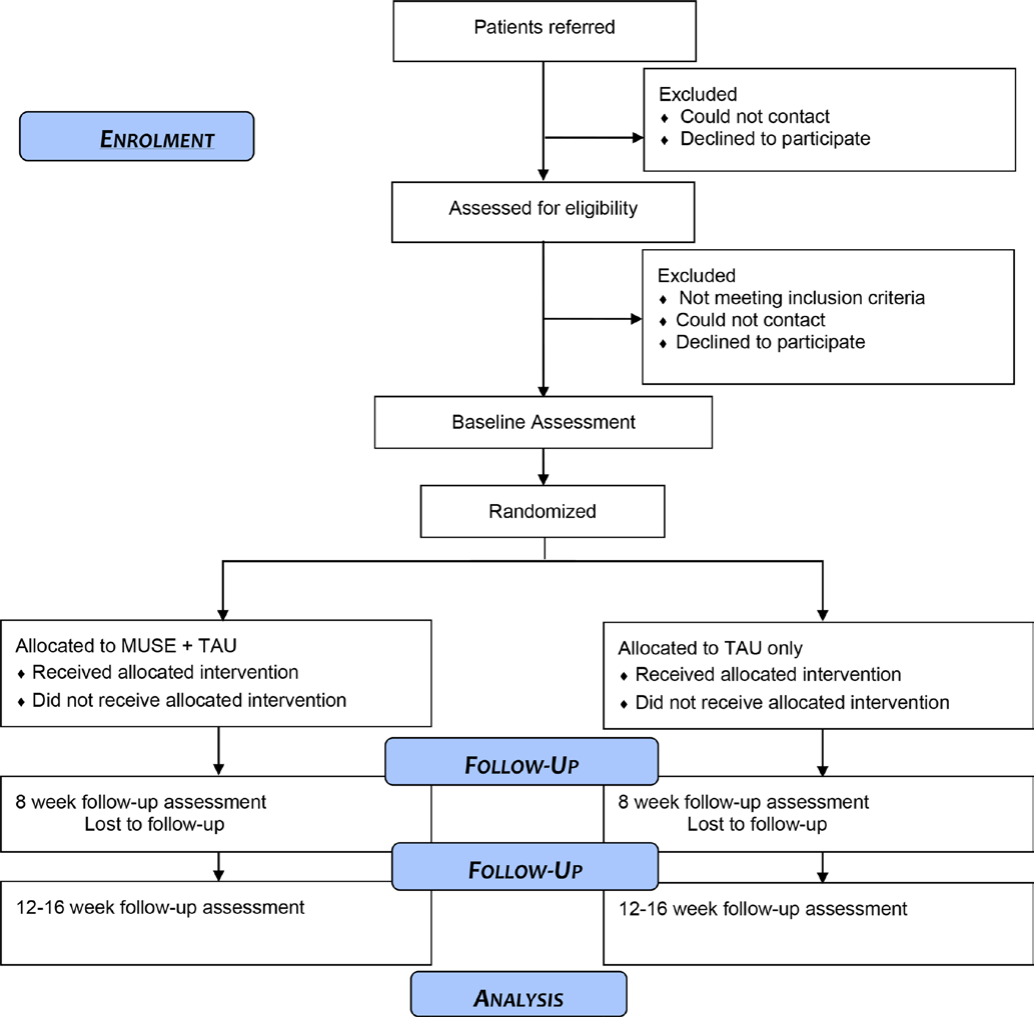
Trial flow diagram. MUSE, Managing Unusual Sensory Experiences; TAU, treatment as usual.

The design is a prospective, single-blind, parallel-group, randomised controlled feasibility trial to evaluate a novel hallucination intervention (MUSE) in addition to usual care versus usual care alone in people with psychosis attending NHS EIP services. Usual care will be recorded but will remain as standard in both groups. Assessments will be carried out at 0 week (baseline), 8 weeks (post-treatment) and 12 weeks (follow-up which is 1 month post therapy but with an allowance of another month if needed) by a researcher blind to treatment allocation. A number of related qualitative studies will explore the acceptability of study procedures, the delivery and experience of the intervention to participants as well as the value of the training and supervision for the staff delivering MUSE. A summary of the trial design can be seen in figure 1. The trial is registered with the ISRCTN registry (registered 24 November 2021). There is an independent trial data monitoring committee (DMC) and a trial steering committee (TSC) and a patient and public involvement (PPI) group facilitated by a coapplicant for the study with lived experience of psychosis.

### Randomisation and blinding

#### Sequence generation

Randomisation to the two groups will be undertaken using the web-based Sealed Envelope randomisation service (https://www.sealedenvelope.com). Randomisation will be in the ratio 1:1 to the two groups and will be stratified by site. Randomisation (at the individual level) will be independent and concealed.

### Concealment mechanism

The randomisation system is web-based and allocation is made known to the trial leads and trial therapists only at the point of randomisation. The allocation is dynamically generated and uses randomly varying blocks of sizes not known to the study team so that allocation concealment is assured.

The researcher undertaking assessments will be blinded to group allocation, but the participants and clinician delivering the intervention cannot be blinded to condition. If there is an unblinding, then future assessments will be undertaken by another assessor to maintain the blind. Breaks in blinding will be monitored and recorded.

### Participants

Participants will be recruited from EIP services in two sites within the UK NHS: Cumbria, Northumberland Tyne and Wear NHS Foundation Trust and Tees, Esk and Wear Valleys NHS Foundation Trust. We will recruit 80 people (aged 16 and over) with distressing hallucinations. Following referral by the clinical team, all people interested in taking part will then be approached by the research team, given information about the trial, and then screened and assessed for inclusion in the trial. Written informed consent will be obtained from each participant prior to any participation.

### Inclusion criteria

Participants will

- Be in contact with EIP services.
- Have an identified care coordinator.
- Meet International Classification of Diseases, 11th Revision, criteria for schizophrenia, schizoaffective disorder or entry criteria for an EIP service.
- Have a history of auditory hallucinations for at least 4 weeks.
- Be aged 16 and above.
- Consider their hallucinations as a main difficulty and would like to receive an intervention specifically for hallucinations.
- Have the capacity to provide informed consent.
- Be judged by their clinician to be clinically stable for the preceding 4 weeks.
- Be both individuals who are on antipsychotic treatment and individuals who decline to take medication, provided no medication changes have occurred in the previous 1 month (ie, having started or stopped antipsychotic medication, or a switch to or from clozapine).

### Exclusion criteria

Participants with the following will not be eligible:

- Hallucinations/psychosis with a known biological basis.
- Insufficient command of English to complete the study procedures.
- Intellectual disability or severe cognitive dysfunction affecting the ability to provide fully informed consent to participate.
- A primary diagnosis of substance misuse/dependency.
- Currently engaged in CBTp or received CBTp in the past 6 months.

### Assessments

Independent assessors blind to therapy group will conduct all assessments at the three time points. Basic demographic and clinical data will be collected (eg, age, gender, ethnicity and clinical diagnosis). Clinical outcomes including hallucinations, affective symptoms, social functioning, quality of life and physical health will be assessed at all three time points (baseline 0 month, postintervention 2 months and follow-up 3–4 months)

Hallucinations are assessed using the Psychotic Symptom Rating Scale,^20^ which is an 11-item semistructured interview, with three items being used to identify voice-related distress. Also, the self-report voice-impact subscale on the Hamilton Programme for Schizophrenia Voices Questionnaire^21^ will also be used. Additional items asking about hallucinations in non-auditory modalities (ie, visual, somatic, olfactory and sense of presence) are assessed as well.^22^

Levels of anxiety and depression (Depression, Anxiety and Stress Scales^23^) as well as perceived recovery (Questionnaire about the Process of Recovery^24^) are assessed. The perceived impact of the intervention (The Choice of Outcome In CBT for Psychoses^25^) is used to assess progress towards therapy-related goals. To determine therapeutic alliance and therapy acceptability, the Satisfaction with Therapy and Therapist Scale^26^ and Working Alliance inventory^27^ are used. In addition, at each session, a short self-assessment form comprising items adapted from the main measure of hallucinations will monitor variations in voice frequency and distress. To help establish health economics, service use and quality of life will be measured by interview/self-report (Short Form-36^28^), EQ-5D,^29^ perceived capability (Investigating Choice Experiments Capability Measure for Adults^30^) and from a case record review using a tool developed for the study.^31^

These measures are to help identify important parameters for a future trial (ie, completion rates and selection of best outcome measures) and to estimate the SD of outcomes in order to facilitate a sample size calculation for use within a definitive trial. Each measure has established psychometric properties for use with this population.

### Managing Unusual Sensory Experiences in People with First-Episode Psychosis (MUSE FEP) intervention

The intervention is a novel digital treatment for hallucinations. The treatment is divided into the following modules:

1. What Are Voices? This module provides normalising information about the frequency of voices and the factors that tend to increase voice hearing (eg, substance misuse and sleep deprivation), along with testimonies from other voice hearers.
2. How the Mind Works. This module outlines current understanding of key psychological processes such as threat detection, the importance of prediction (top-down processing) and how intrusive thoughts work.
3. Assessment. This module identifies the subtype of hallucination a service user is experiencing (inner speech–auditory verbal hallucination (AVH), a memory-based AVH or a hypervigilance AVH).
4. Inner Speech. This module provides psychoeducation about the evidence that voice hearing involves people not recognising their own inner speech. An individual understanding or formulation of voice-hearing experiences is coproduced, and then targeted coping strategies and behavioural experiments are employed, such as means of interrupting and manipulating inner speech via singing or humming.
5. Memory and Trauma. This module provides psychoeducation about how memories from traumatic events are more likely to be experienced as intrusive memories without contextual cues and can therefore be experienced as belonging to the here and now. Treatment helps people manage and reframe difficult memories.
6. Hypervigilance. This module describes how our brain uses prediction to interpret the world and make sense of sensory data received. If people are expecting threatening stimuli, they may struggle to scrutinise poor quality sensory data and rely more heavily on predictions while adopting a ‘better safe than sorry’ decision bias. Based on this understanding, targeted coping strategies are employed (such as reducing arousal and stress when under threat).
7. Seeing Visions. This module draws on these other modules and explains how our visual perceptual system can lead to mistaken perceptions, for example, how easily we see faces in clouds. An individual formulation and treatment plan is then developed that normalises the experience and addresses the key cause of distress, and then targeted coping strategies and behavioural experiments are employed (such as training oneself to switch attention to and from visions).
8. Sleep. This module provides psychoeducation and treatment strategies about sleep, which is often a key factor in all types of unusual sensory experiences.

The intervention is delivered by a care coordinator on an individual basis in up to eight 1-hour sessions, though four to six is the usual number. There is typically one session per week. A treatment dose is defined as three or more sessions. MUSE is provided flexibly, often in people’s homes or clinics. MUSE is provided in addition to usual care. With patient consent, sessions will be audio-recorded and independently rated for quality, including fidelity and competence. MUSE is provided by care coordinators who have attended a 3-day training in MUSE. They are offered regular supervision sessions. As a minimum, we aim to train one team member per service that we recruit from, and they would provide the intervention in that service.

### Control condition

The control condition is treatment as usual which is support from EIP services. Each participant will have a care coordinator who will see the person weekly in their home usually for around 30–60 min. Where appropriate, patients are offered low-dose antipsychotic medication, psychological therapies (CBTp and family interventions), social support and recovery-based activities and help to manage their symptoms (but not MUSE). As per our exclusion criteria, we would not accept someone currently receiving CBTp, but past the recruitment stage, we will not be asking referrers to withhold any treatment. A case report form will be used to track treatment as usual (TAU) content throughout the trial based on the Template for Intervention Description and Replication checklist.^31^ The MUSE intervention group will also receive TAU.

### Analysis

A full statistical analysis plan will be finalised before any analysis takes place. Analyses will be based on the intention-to-treat approach. Data will be reported in line with the Consolidated Standards of Reporting Trials–Social and Psychological Interventions statement and Standard Protocol Items Recommendations for Interventions Trial SPIRIT guidelines. This guidance recommends minimising the distinction between primary and secondary outcomes, so all outcomes will be reported at the end of the trial.

### Sample size

As the main objective was to establish feasibility parameters for a definitive trial, there are no formal sample size calculations, interim analyses or stopping rules. We have estimated attrition of 12.5% based on past research of psychological therapy with people with psychosis^32^ and similar brief interventions,^33^ meaning 70 people will complete the study. Guidance on external pilot studies indicates that samples of 35 per arm or more give a reliable estimate of the SD of the outcome measure.^34 35^

### Statistical analysis

Descriptive statistics within each randomised group will be presented for baseline, end of treatment and follow-up values. These will include frequency and percentages for binary and categorical variables, and means and SD, or medians with IQR, for continuous variables, along with minimum and maximum values, and frequency and percentages of missing values. There will be no tests of statistical significance or CIs for differences between randomised groups on any baseline variable.

We will analyse change scores for the quantitative measures used across assessments using linear mixed effects models adjusted for site to estimate effect size between MUSE+TAU and TAU alone. The choice of mixed effect models will address the repeated measure data structure at 2 and 3 months. These will be used to inform the overall interpretation of participants’ experience of therapy (combining qualitative and quantitative data). We are also interested in understanding which areas of the participant’s life the intervention may impact on, for example, distress linked to voice hearing, interpersonal functioning, etc.

### Qualitative analysis

Qualitative data, gathered from the structured interviews with patients and staff, will be analysed using an inductive thematic analysis.^36^ This form of analysis allows for both basic and more complex themes to be classified from interview data (based on categorising sections of transcripts), based on the data themselves rather than a prior theory.^37^

### Health economics analysis

For a future health economic analysis, we will determine the acceptability and completeness of the necessary data (care records tool, medication use, ICEpop CAPability measure ICECAP and EQ-5D).

### Criteria for proceeding to a full trial

We will use criteria for assessing study success and identifying feasibility factors required for delivering the definitive study^38^ and follow A Systematic Process for Decision Making after Pilot and Feasibility Trials (ADePT^39^), which helps identify criteria used to go to a full trial. These criteria are being developed with the PPI group to help determine if a full trial is warranted. We will use criteria on participant recruitment, adherence with the intervention and retention at follow-up to assess the trial (as set out in ADePT), plus data on uptake, retention of participants, intervention fidelity and acceptability. This will use both quantitative and qualitative data derived from the study.^40^ The progression criteria will be divided into three categories (green, red and amber^38^). Areas that are amenable to change before a pilot trial will be investigated and solutions will be discussed with the patient group for acceptability. This will help consider if a full trial is timely, necessary and deliverable.^35 41^

### Adverse events

Adverse events will be reported for all participants randomised. A serious adverse event (SAE) is defined as any untoward medical occurrence that results in death, is life threatening, requires or prolongs hospitalisation, or results in persistent or significant disability/incapacity. Accordingly, we will record admissions to hospital, suicide attempts, serious violent incidents and deaths. Treatment or hospitalisation for a pre-existing physical health condition, which has not worsened, does not constitute an SAE. We will record the occurrence of any SAEs reported to us and also systematically check all participants’ medical records following completion of the final assessment. We will also record formal complaints regarding therapy. The relevant clinical team, sponsor and DMC will be informed of any adverse event. The DMC chair and sponsor will determine if it is trial related.

### Patient and public involvement

PPI is facilitated by a coapplicant with personal experience of psychosis. The grant application and MUSE intervention were developed in collaboration with people who have lived with hallucinations. Following the award of the grant, a fully funded PPI group has been formed in line with recommendations of advisory groups that encourage service-user involvement in NHS research (http://www.invo.org.uk/). The PPI group will be actively involved in all stages of the research programme, for example, advising on participant recruitment, information sheets, staff training, topic guides, analysis of the qualitative interview data and dissemination of the research findings. PPI members also sit on the TSC.

## Ethics and dissemination

The trial obtained ethical approval from the NHS Yorkshire and the Humber-Sheffield Research Ethics Committee (21/YH/0090) and Health Research Authority(IRAS 292150, the MUSE FEP trial). CNTW NHS Foundation Trust is the trial sponsor. The R&D team in the two recruitment sites (Cumbria, Northumberland, Tyne and Wear, CNTW and Tees, Esk and Wear Vally, TEWV NHS Trusts) have confirmed local capacity and capability to deliver the research. The results of the trial will be published in a peer-reviewed journal and made open access. On the basis of a reasonable request, the main outcome data (anonymised) will be available from the trial team after publication of the main results paper. The PPI team will help to provide feedback to all the participants, staff and services involved in the study.

### Trial status

The trial started recruitment in June 2021 and will last for 12 months until May 2022, with final outcome data collected by September 2022. A trial paper detailing the outcomes should be submitted for publication around December 2022.

## Supplementary Material

Reviewer comments

Author's manuscript
