# Supplementary material for: Managing Unusual Sensory Experiences in People with First-Episode Psychosis (MUSE FEP): a study protocol for a single-blind parallel-group randomised controlled feasibility trial
Source: BMJ Open. 2022 May 16;12(5):e061827. doi: 10.1136/bmjopen-2022-061827 (PMC9114953; doi:10.1136/bmjopen-2022-061827)
Supplement: Author's manuscript [file bmjopen-2022-061827.draft_revisions.pdf]

BMJ Open is committed to open peer review. As part of this commitment we make the peer review history of every article we publish publicly available.

When an article is published we post the peer reviewers' comments and the authors' responses online. We also post the versions of the paper that were used during peer review. These are the versions that the peer review comments apply to.

The versions of the paper that follow are the versions that were submitted during the peer review process. They are not the versions of record or the final published versions. They should not be cited or distributed as the published version of this manuscript.

BMJ Open is an open access journal and the full, final, typeset and author-corrected version of record of the manuscript is available on our site with no access controls, subscription charges or pay-per-view fees (http://bmjopen.bmj.com).

If you have any questions on BMJ Open's open peer review process please email info.bmjopen@bmj.com

BMJ Open

Managing Unusual Sensory Experiences (MUSE) in people with first episode psychosis: A study protocol for a single blind parallel group randomised controlled feasibility trial (MUSE FEP).

Journal:	*BMJ Open*
Manuscript ID	bmjopen-2022-061827
Article Type:	Protocol
Date Submitted by the Author:	08-Feb-2022
Complete List of Authors:	dudley, robert; Newcastle University; Cumbria Northumberland Tyne and Wear NHS Foundation Trust<br>Dodgson, Guy; Cumbria Northumberland Tyne and Wear NHS Foundation Trust<br>Common, Stephanie; Tees Esk and Wear Valleys NHS Foundation Trust<br>O'Grady, Lucy; Cumbria Northumberland Tyne and Wear NHS Foundation Trust<br>Watson, Florence; Tees Esk and Wear Valleys NHS Foundation Trust<br>Gibbs, Christopher; Cumbria Northumberland Tyne and Wear NHS Foundation Trust<br>Arnott, Bronia; Newcastle University Population Health Sciences Institute<br>Fernyhough, Charles; University of Durham, Department of Psychology<br>Alderson-Day, B; Durham University,<br>Ogundimu, Emmanuel; University of Durham<br>Kharatikoopaei, Ehsan; University of Durham<br>Patton, Victoria; University of Durham<br>Aynsworth, Charlotte; Cumbria Northumberland Tyne and Wear NHS Foundation Trust
Keywords:	Schizophrenia & psychotic disorders < PSYCHIATRY, Adult psychiatry < PSYCHIATRY, Clinical trials < THERAPEUTICS

SCHOLARONE™
Manuscripts

Managing Unusual Sensory Experiences (MUSE) in people with first episode psychosis: A study protocol for a single blind parallel group randomised controlled feasibility trial (MUSE FEP).

Robert Dudley*[1,2], Guy Dodgson[1], Stephanie Common[3], Lucy O'Grady[1], Florence Watson[3], Christopher Gibbs[1], Bronia Arnott[2], Charles Fernyhough[4], Ben Alderson-Day[4], Emmanuel Ogindimu[5], Ehsan Kharatikoopaei [5], Victoria Patton[4], and Charlotte Aynsworth[1].

1 Cumbria, Northumberland, Tyne and Wear NHS Foundation Trust, St. Nicholas Hospital, Jubilee Road, Gosforth, Newcastle Upon Tyne, NE3 3XT, United Kingdom.

2 Population Health Sciences Institute, Faculty of Medical Sciences, Newcastle University, Baddiley Clark Building, Richardson Road, Newcastle Upon Tyne, NE1 4AX, United Kingdom.

3 Tees, Esk & Wear Valley NHS Trust, Wessex House, Falcon Court, Stockton on Tees, TS18 3TX, United Kingdom.

4. Caedmon Building, Leazes Road, University of Durham, Durham, DH1 1SZ, United Kingdom.

5. University of Durham, Stockton Road, Durham, DH1 3LE, United Kingdom.

Word count excluding title page, abstract, references and figures 3897

* Address for correspondence: Dr Robert Dudley, Gateshead Early Intervention in Psychosis service, Dryden Centre, Evistones Road, Low Fell, Gateshead, United Kingdom, NE9 5UR.

Tel 0191 223 2020 email rob.dudley@cntw.nhs.uk

For peer review only

ABSTRACT

Introduction Hallucinations (hearing or seeing things that others do not) are a common feature of psychosis, causing significant distress and disability. Existing treatments such as Cognitive Behaviour Therapy for psychosis (CBTp) have modest benefits and there is a lack of CBTp-trained staff. Shorter, targeted treatments that focus on specific symptoms delivered by a non-specialist workforce could substantially increase access to treatment.

MUSE (Managing Unusual Sensory Experiences) explains why people have hallucinations and helps the person to develop and use coping strategies to reduce distress. MUSE focuses only on hallucinations, and treatment is short (4-6, one hour weekly sessions). It is a digital intervention, run on NHS laptops, which provides information about hallucinations in an engaging way, using audio, video, and animated content. Crucially, it is designed for use by non-specialist staff like Community Psychiatric Nurses (CPNs).

Methods and analysis The study is a two-arm feasibility Randomised Controlled Trial comparing MUSE and treatment as usual (TAU) (n=40) to TAU alone (n=40), recruiting across two NHS Trusts, using 1:1 allocation and blind assessments before and after treatment; (2 months), and at follow up (3 months). Quantitative information on recruitment rates, adherence, and completion of outcome assessments will be collected. Qualitative interviews will capture service-users' experience of therapy and clinicians' experiences of the training and supervision in MUSE. Clinicians will also be asked about factors affecting uptake, adherence, and facilitators/barriers to implementation. Analyses will focus on feasibility outcomes and provide initial estimates of intervention effects. Thematic analysis of the qualitative interviews will assess the acceptability of the training, intervention, and trial procedures.

For peer review only - http://bmjopen.bmj.com/site/about/guidelines.xhtml

Ethics and dissemination The trial has received NHS Ethical and Health Research Authority approval. Findings will be disseminated directly to participants, and services as well as through peer-reviewed publications and conference presentations.

Trial registration number ISRCTN16793301

For peer review only

For peer review only - http://bmjopen.bmj.com/site/about/guidelines.xhtml

Strengths and limitations

The MUSE FEP trial is the first digital treatment for distressing hallucinations delivered by frontline staff.

PPI involvement has been woven in throughout the development of the treatment and in the design and running of the study.

This randomised controlled multisite single-blind trial targets proposed causal factors implicated in the development of hallucinations.

The follow-up period is short, meaning that we cannot determine whether any benefits endure.

There is no active control, so we do not know what factors contribute to any change.

For peer review only

BACKGROUND

Almost all people with psychosis at some stage report hallucinations (hearing or seeing things that others do not[1]. Whilst for some these experiences can be positive[2] for many others these hallucinations can be distressing and disabling[3]. To address this need, treatments like Cognitive Behavioural Therapy for Psychosis (CBTp) are recommended[4]. A recent meta-analysis[5] indicated that CBTp has a specific and beneficial effect on hallucinations in comparison to active and inactive control conditions (Hedges g=0.34). Whilst welcome, the effects are still relatively modest. Moreover, access to CBTp remains limited[6] owing to the number of therapists available and the time it requires to deliver treatment[7]. CBTp is meant to be offered as 16–24 weekly sessions offered over 6-9 months.

Two approaches can help. The first is to provide briefer interventions allowing more people to receive treatment. However, the benefits of CBTp are already modest and dose of therapy is associated with outcome[8]. To retain the impact in a briefer form it is essential that shorter treatments are sufficiently potent, which will likely involve targeting key causal mechanisms[9] leading to hallucinations. The second approach is to increase access by enabling a larger and less well-trained workforce to provide treatment. The present study combines these approaches.

In terms of causal mechanisms, the exact reasons people see and hear things others do not is unclear. However, research on auditory hallucinations has shown that they take different forms that may respond to specific therapeutic approaches. A review by the International Consortium on Hallucinations Research[10] outlined at least three different potential routes to experiencing voices for people with psychosis which may be suited to different kinds of tailored intervention: *inner speech* voices (postulated to result from the misattribution of ordinary inner speech to an external agent); *hypervigilance* voices (resulting

For peer review only

from a biased attention to environmental stimuli); *memory* voices (the result of intrusions from traumatic memory[11]).

The present approach draws on this understanding and utilises it in a novel, digital toolkit called MUSE (Managing Unusual Sensory Experiences) that focuses only on hallucinations. MUSE uses psychoeducation about the currently known causal mechanisms of hallucination as means of exploring, with service-users, why their specific experiences may be happening. This knowledge is then matched to specific, tailored interventions and coping strategies that enable the person to understand manage their experiences differently and reduce their distress. This process relies on psychoeducation as its basis, but it is more fundamentally about helping the person to change their understanding, manage their experiences better, and thus cope more effectively. MUSE is a departure from traditional CBTp as it focuses only on hallucinations and reduces the typical number of sessions to 4–6. This helps prevent the "drift" that can occur in treatment when there is not a specific target symptom or clear conceptual model underpinning treatment.

To help increase access to therapy, attempts have been made to train non-psychological therapy staff such as Community Psychiatric Nurses (CPNs) in the delivery of talking therapies[12]. However, such efforts have not demonstrated the anticipated benefits, owing to the challenge of managing high caseloads, and of providing brief training for a complex intervention and a lack of standardisation of delivery[13,14]). Recently, brief interventions (defined as less than 12 sessions[15]) provided by Assistant Psychologists for hallucinations have been trialled with some encouraging findings[16] indicating that brief targeted therapies can be provided by non-psychological therapists.

The present research continues to address this unmet need by putting high-quality treatment tools in the hands of Care Coordinators working in Early Intervention in Psychosis services (EIP). Care Coordinators (who are often Community Psychiatric Nurses (CPNs)) are

For peer review only - http://bmjopen.bmj.com/site/about/guidelines.xhtml

the most widely available workforce in EIP teams[6] potentially providing the maximum access to high-quality care. EIP care coordinators work with smaller case-loads (12-15) than in other services meaning there is capacity to deliver the treatment. Utilising the benefits of digital technology, MUSE is manualised and loaded onto an NHS laptop meaning that treatment is standardised, reducing the training required and the risk that staff will not feel confident in delivery. MUSE presents the content in a user-friendly, engaging way and is designed for joint use of therapist and service-user in the therapy session. Clinicians use prepared videos and other types of media to help explain why the person may see or hear things others do not, and to help service-users to practise coping strategies and carry out behavioural experiments to test the reality of their experiences.

An earlier version of MUSE was delivered to people with distressing voices by psychological therapists (not Care Coordinators who will deliver the toolkit in this project) in a recently completed uncontrolled single group feasibility study (MUSE PSYCHOSIS[17]). Twelve participants who completed follow-up after 10 MUSE sessions reported high treatment satisfaction and acceptability (87% & 90% mean overall ratings). The within-subject effect size for reducing auditory hallucination severity was large (d=0.7). Even greater benefits were evident on a recently completed uncontrolled study in which MUSE was used by psychological therapists with a group of people in an At Risk Mental State (ARMS) for psychosis[18]. The effect size was d=0.8 (n=19) for reduction of the intensity of auditory hallucinations, and satisfaction ratings were 90%. These studies provide encouraging evidence of the value of this approach when delivered by expert therapists.

Learning from these studies the toolkit was revised in three important ways. First, MUSE now incorporates more information about a range of hallucinations and not just auditory[19]. Second, the lived-experience input was strengthened to improve the relevance and accessibility of the MUSE materials. Third, MUSE was made easier to use for frontline

For peer review only - http://bmjopen.bmj.com/site/about/guidelines.xhtml

staff and specialist training and supervision for these staff was developed to enable them to use the MUSE toolkit.

The treatment now requires testing in a randomised trial to determine the effects on hallucinations and whether access can be improved if delivered by a non-specialist workforce. Consequently, the present study investigates the feasibility and acceptability of MUSE treatment delivered by CPNs in participants experiencing first-episode psychosis.

Objectives

The primary objective of the present study is to establish if it is possible to undertake a larger, definitive trial in the future. Therefore, we will collect data to inform the feasibility and acceptability of a targeted hallucination-focussed treatment delivered by non-psychological therapists to establish the key parameters for a future definitive trial. The study will use mixed methods to i) assess the feasibility of CPN staff training and delivery of the MUSE toolkit; ii) asses the acceptability of the MUSE toolkit to patients and staff; and to iii) collect data to inform the effectiveness of future definitive trial (including referral, recruitment, uptake and attrition rates).

METHODS AND ANALYSIS

Trial design and flow chart

The design is a prospective, parallel group, single-blind, randomised controlled feasibility trial to evaluate a novel hallucination intervention (MUSE) in addition to usual care versus usual care alone in people with psychosis attending NHS EIP services. Usual care will be recorded, but remain as standard in both groups. Assessments will be carried out at 0 (baseline), 8 weeks (post-treatment) and 12 weeks (follow-up which is 1m post-therapy but

For peer review only - http://bmjopen.bmj.com/site/about/guidelines.xhtml

with an allowance of another month if needed) by a researcher blind to treatment allocation. A number of related qualitative studies will explore the acceptability of study procedures, the delivery and experience of the intervention to participants as well as the value of the training and supervision for the staff delivering MUSE. A summary of the trial design can be seen in figure 1. The trial is registered with the ISRCTN registry: 16793301(registered 7/12/2021). There is an independent trial Data Monitoring Committee (DMC) and a Trial Steering Committee (TSC) and Patient Public Involvement group (PPI) facilitated by a co-applicant for the study with lived experience of Psychosis.

Figure one about here please

Randomisation and blinding

Sequence generation

Randomisation to the two groups will be undertaken using the web-based Sealed Envelope randomisation service (https://www.sealedenvelope.com). Randomisation will be in the ratio 1:1 to the two groups and will be stratified by site. Randomisation (at the individual level) will be independent and concealed.

Concealment mechanism

The randomization system is web-based and allocation is made known to the trial leads and trial therapists only at the point of randomisation. The allocation is dynamically generated and uses randomly varying blocks of sizes not known to the study team, so that allocation concealment is assured.

For peer review only

The researcher undertaking assessments will be blinded to group allocation, but the participants and clinician delivering the intervention cannot be blinded to condition. If there is an unblinding then future assessments will be undertaken by another assessor to maintain the blind. Breaks in blinding will be monitored and recorded.

Participants

Participants will be recruited from EIP services in two sites within the UK National Health Service (NHS) – Cumbria, Northumberland Tyne and Wear NHS Foundation Trust and Tees, Esk and Wear Valleys NHS Foundation Trust. We will recruit 80 people (aged 16 and over) with distressing hallucinations. Following referral by the clinical team, all people interested in taking part will then be approached by the research team, given information about the trial, and then screened and assessed for inclusion in the trial. Written informed consent will be obtained from each participant prior to any participation.

Inclusion criteria

Participants will:

- Be in contact with Early Intervention in Psychosis (EIP) services.
- Have an identified care coordinator.
- Meet ICD-11 criteria for schizophrenia, schizoaffective disorder or entry criteria for an EIP service.
- Have a history of auditory hallucinations for at least four weeks.
- Be aged 16 and above.
- Consider their hallucinations as a main difficulty, and would like to receive an intervention specifically for hallucinations.
- Have the capacity to provide informed consent.

For peer review only

- Be judged by their clinician to be clinically stable for the preceding 4 weeks.
- Both individuals on antipsychotic treatment, and those who decline to take medication, will be included, provided no medication changes have occurred in previous 1m (i.e., having started or stopped antipsychotic medication, or a switch to or from Clozapine).

Exclusion criteria

Participants with the following will not be eligible:

- Hallucinations/psychosis with a known biological basis.
- Insufficient command of English to complete the study procedures.
- Intellectual disability, or severe cognitive dysfunction affecting the ability to provide fully informed consent to participate.
- A primary diagnosis of substance misuse/dependency.
- Currently engaged in CBTp or received CBTp in the past 6 months.

Assessments

Independent assessors blind to therapy group will conduct all assessments at the three time points. Basic demographic and clinical data will be collected (e.g., age, gender, ethnicity, clinical diagnosis). Clinical outcomes including hallucinations, affective symptoms, social functioning, quality of life and physical health will be assessed at all three time points (baseline 0 months, post-intervention 2 months and follow-up 3-4 months)

Hallucinations are assessed using the Psychotic Symptom Rating Scales[20] which is an 11 item semi-structured interview, with 3 items being used to identify voice related distress. Also, the self-report voice-impact subscale on the Hamilton Program for Schizophrenia Voices Questionnaire (HPSVQ)[21] will also be used. Additional items asking

For peer review only

about hallucinations in non-auditory modalities (i.e., visual, somatic, olfactory and sense of presence) are assessed as well[22].

Levels of anxiety and depression (Depression, Anxiety and Stress Scales (DASS[23]) as well as perceived recovery (QPR process of recovery questionnaire[24]) are assessed. The perceived impact of the intervention (The CHoice of Outcome In Cbt for psychosEs (CHOICE[25]) is used to assess progress towards therapy-related goals. To determine therapeutic alliance & therapy acceptability, the Satisfaction with Therapy and Therapist Scale[26]) and Working Alliance inventory[27] are used. In addition, at each session, a short self-assessment form comprising items adapted from the main measure of hallucinations will monitor variations in voice frequency and distress. To help establish health economics, service use and quality of life will be measured by interview/self report (Short Form-36[28]), EQ-5D[29], perceived capability (Investigating Choice Experiments Capability Measure for Adults (ICECAP-A[30]) and from a case record review using a tool developed for the study[31].

These measures are to help identify important parameters for a future trial (i.e. completion rates and selection of best outcome measures) and to estimate the standard deviation of outcomes in order to facilitate a sample-size calculation for use within a definitive trial. Each measure has established psychometric properties for use with this population.

The MUSE FEP intervention

The intervention is a novel digital treatment for hallucinations. The treatment is divided into the following Modules:

For peer review only - http://bmjopen.bmj.com/site/about/guidelines.xhtml

1. *What are Voices?* This module provides normalising information about the frequency of voices and the factors that tend to increase voice-hearing (for example, substance misuse and sleep deprivation), along with testimonies from other voice-hearers.

2. *How the Mind Works*. This module outlines current understanding of key psychological processes such as threat detection, the importance of prediction (top-down processing) and how intrusive thoughts work.

3. *Assessment.* This module identifies the subtype of hallucination a service-user is experiencing (Inner Speech-Auditory verbal hallucination (AVH), a Memory Based AVH or a Hypervigilance AVH).

4. Inner Speech. This module provides psycho-education about the evidence that voice-hearing involves people not recognising their own inner speech. An individual understanding or formulation of voice-hearing experiences is co-produced and then targeted coping strategies and behavioural experiments are employed, such as means of interrupting and manipulating inner speech via singing or humming.

5. Memory and Trauma. This module provides psycho-education about how memories from traumatic events are more likely to be experienced as intrusive memories without contextual cues, and can therefore be experienced as belonging to the here and now. Treatment helps people manage and reframe difficult memories.

6. Hypervigilance. This module describes how our brain uses prediction to interpret the world and make sense of sensory data received. If people are expecting threatening stimuli they

For peer review only

may struggle to scrutinise poor quality sensory data and rely more heavily on predictions, whilst adopting a 'better safe than sorry' decision bias. Based on this understanding targeted coping strategies are employed (such as reducing arousal and stress when under threat).

7. Seeing Visions. This module draws on these other modules, and explains how our visual perceptual system can lead to mistaken perceptions, for example how easily we see faces in clouds. An individual formulation and treatment plan is then developed that normalises the experience and addresses the key cause of distress and then targeted coping strategies and behavioural experiments are employed (such as training oneself to switch attention to and from visions).

8. Sleep. This module provides psycho-education and treatment strategies about sleep, which is often a key factor in all types of unusual sensory experiences.

The intervention is delivered by a Care Coordinator on an individual basis in up to eight 1-hour sessions, though 4-6 is the usual number. There is typically one session per week. A treatment dose is defined as three or more sessions. MUSE is provided flexibly, often in people's homes or clinics. MUSE is provided in addition to usual care. With patient consent, sessions will be audio-recorded and independently rated for quality, including fidelity and competence. MUSE is provided by care coordinators who have attended a three-day training in MUSE. They are offered regular supervision sessions. As a minimum we aim to train one team member per service that we recruit from, and they would provide the intervention in that service.

Control condition

For peer review only - http://bmjopen.bmj.com/site/about/guidelines.xhtml

The control condition is treatment-as-usual which is support from EIP services. Each participant will have a Care Coordinator, who will see the person weekly in their home usually for around 30 to 60 minutes. Where appropriate, patients are offered low dose antipsychotic medication, psychological therapies (CBTp and Family Interventions), social support, and recovery-based activities and help to manage their symptoms (but not MUSE). As per our exclusion criteria we would not accept someone currently receiving CBTp, but past the recruitment stage we will not be asking referrers to withhold any treatment. A case report form will be used to track TAU content throughout the trial based on the TIDieR checklist[31]. The MUSE intervention group will also receive TAU.

Analysis

A full statistical analysis plan will be finalised before any analysis takes place. Analyses will be based on the intention-to-treat approach. Data will be reported in line with the CONSORT - Social and Psychological Interventions (CONSORT-SPI) statement, and SPIRIT guidelines. This guidance recommends minimising the distinction between primary and secondary outcomes so all outcomes will be reported at the end of the trial.

Sample size

As the main objective is to establish feasibility parameters for a definitive trial there are no formal sample size calculations, interim analyses or stopping rules. We have estimated attrition of 12.5% based on past research of psychological therapy with people with psychosis[32] and similar brief interventions[33] meaning 70 people will complete the study. Guidance on external pilot studies indicates that samples of 35 per arm or more give a reliable estimate of the standard deviation of the outcome measure[34,35].

Statistical analysis

For peer review only - http://bmjopen.bmj.com/site/about/guidelines.xhtml

Descriptive statistics within each randomised group will be presented for baseline, end of treatment and follow up values. These will include frequency and percentages for binary and categorical variables, and means and standard deviations, or medians with interquartile range (QR), for continuous variables, along with minimum and maximum values, and frequency and percentages of missing values. There will be no tests of statistical significance or confidence intervals for differences between randomised groups on any baseline variable.

We will analyse change scores for the quantitative measures used across assessments using linear mixed effects models adjusted for site to estimate effect size between MUSE+TAU and TAU alone. The choice of mixed effect models will address the repeated measure data structure at 2 and 3 months. These will be used to inform the overall interpretation of participants' experience of therapy (combining qualitative and quantitative data). We are also interested in understanding which areas of the participant's life the intervention may impact upon, for example, distress linked to voice hearing, interpersonal functioning etc.

Qualitative analysis

Qualitative data, gathered from the structured interviews with patients and staff, will be analysed using an inductive thematic analysis[36]. This form of analysis allows for both basic and more complex themes to be classified from interview data (based on categorising sections of transcripts), based on the data themselves rather than a prior theory[37].

Health economics analysis

For a future health economic analysis we will determine the acceptability and completeness of the necessary data (care records tool, medication use, ICECAP and EQ-5D).

For peer review only - http://bmjopen.bmj.com/site/about/guidelines.xhtml

Criteria for proceeding to a full trial

We will use criteria for assessing study success and identifying feasibility factors required for delivering the definitive study [38] and follow a systematic process for decision making after pilot and feasibility trials (ADePT[39]) which helps identify criteria used to go to a full trial. These criteria are being developed with the PPI group to help determine if a full trial is warranted. We will use criteria on participant recruitment, adherence with the intervention, and retention at follow-up to assess the trial (as set out in ADePT), plus data on uptake, retention of participants, intervention fidelity and acceptability. This will use both quantitative and qualitative data derived from the study[40]. The progression criteria will be divided into three categories (green, red and amber[38]). Areas that are amenable to change before a pilot trial will be investigated and solutions discussed with the patient group for acceptability. This will help consider if a full trial is timely, necessary, and deliverable[41,42].

Adverse events

Adverse events will be reported for all participants randomised. A serious adverse event (SAE) is defined as any untoward medical occurrence that results in death, is life threatening, requires or prolongs hospitalisation or results in persistent or significant disability/incapacity. Accordingly, we will record admissions to hospital, suicide attempts, serious violent incidents and deaths. Treatment or hospitalisation for a pre-existing physical health condition, which has not worsened, does not constitute an SAE. We will record the occurrence of any SAEs reported to us and also systematically check all participants' medical records following completion of the final assessment. We will also record formal complaints regarding therapy.

For peer review only - http://bmjopen.bmj.com/site/about/guidelines.xhtml

The relevant clinical team, sponsor, and DMC will be informed of any adverse event. The DMC Chair and Sponsor will determine if it is trial related.

Patient and public involvement

Patient and Public Involvement (PPI) is facilitated by a co-applicant with personal experience of psychosis. The grant application and MUSE intervention were developed in collaboration with people who have lived with hallucinations. Following the award of the grant, a fully funded PPI group has been formed in line with recommendations of advisory groups that encourage service-user involvement in NHS research (http://www.invo.org.uk/). The PPI group will be actively involved in all stages of the research programme, for example, advising on participant recruitment, information sheets, staff training, topic guides, analysis of the qualitative interview data and dissemination of the research findings. PPI members also sit on the TSC.

Ethics and dissemination

The trial received ethical approval from the NHS Yorkshire and the Humber-Sheffield Research Ethics Committee (21/YH/0090) Health Research Authority (HRA/ HCRW) approval (IRAS 292150, The MUSE FEP trial). CNTW NHS Foundation Trust is the trial sponsor. The R&D team in the two recruitment sites (CNTW and TEWV NHS trusts) have confirmed local capacity and capability to deliver the research. The results of the trial will be published in a peer-reviewed journal and made open access. On the basis of a reasonable request the main outcome data (anonymised) will be available from the trial team after publication of the main results paper. The PPI team will help to provide feedback to all the participants, staff and services involved in the study.

For peer review only

For peer review only - http://bmjopen.bmj.com/site/about/guidelines.xhtml

Trial status

The trial started recruitment in June 2021 and will last for 12 months until May 2022, with final outcome data collected by September 2022. A trial paper detailing the outcomes should be submitted for publication around December 2022.

Funding statement

This paper presents independent research funded by the NIHR under its Research for Patient Benefit (RfPB) Programme (Grant Reference Number: NIHR201078). MUSE was developed with support from the Wellcome Trust (grant number WT1087201). The study sponsor and funder have no role in study design; collection, management, analysis, and interpretation of data; writing of the report; and the decision to submit the report for publication.

Competing interests

RD receives payment for workshops in treating hallucinations and GD and RD declare they are involved in running treatment studies investigating psychological therapies for psychosis. All other authors declare no competing interest. MUSE is not licensed and will be made freely available if proven to be beneficial.

Acknowledgements

We express our thanks to members of the PPI Panel for their contributions to the design of the trial and development of this protocol. We thank Adetayo Kasim and Professor Luke Vale for their input to the statistics and health economics. Kaja Mitrenga helped with set up of the study and literature reviewing. Professor Sandra Bucci, Dr. Alison Brabban, and Dr. Valentina Short are supporting the DMC and TSC.

For peer review only

Contributors RD is the chief investigator, had overall responsibility for the trial design and drafted the trial protocol. CF, BAD, BA, CG, GD, SC, contributed to the study design. GD, SC, BAD and CF led the development of the treatment with substantial input from a range of clinicians and service users. CG is responsible for Patient and Public Involvement, with support from VP. EK and EO are responsible for the statistical analysis. CA, FW and LOG are co-ordinating the trial and are responsible for recruitment. All authors read and approved the final trial protocol.

For peer review only

References

1. McCarthy-Jones S, Smailes D, Corvin A, et al. Occurrence and co-occurrence of hallucinations by modality in schizophrenia spectrum disorders. *Psychiatry Res.*, 2017; 252, 154-60.
2. Beavan V, Read J, Cartwright C. The prevalence of voice-hearers in the general population: a literature review. *J Ment Health.* 2011;20:281–92.
3. Morrison AP, Nothard S, Bowe SE et al. Interpretations of voices in patients with hallucinations and non-patient controls: a comparison and predictors of distress inpatients. *Behav Res Ther*. 2004;42:1315–23.
4. National Institute for Clinical Excellence. Psychosis and schizophrenia in adults: prevention and management (CG178). NICE, 2014.
5. Turner DT, Burger S, Smit F, et al. What Constitutes Sufficient Evidence for Case Formulation-Driven CBT for Psychosis? Cumulative Meta-analysis of the Effect on Hallucinations and Delusions. *Schizophr Bull.* 2020 Mar 27;46(5):1072–85.
6. The Royal College of Psychiatrists. National Clinical Audit of Psychosis – National Report for the Core Audit. London: Healthcare Quality Improvement Partnership; 2018. https://www.hqip.org.uk/wp-content/ uploads/2018/07/NCAP-National-report-for-core-audit-2018-FINAL.pdf. .
7. Ince P, Haddock G, Tai S. A systematic review of the implementation of recommended psychological interventions for schizophrenia: rates, barriers, and improvement strategies. *Psychol Psychother.* 2016;89:324–50.
8. Spencer H, Dudley R, Freeston M, et al. What are the essential ingredients of a CBT case conceptualization for voices and delusions in schizophrenia spectrum disorders? A study of expert consensus. *Schizophr Res*. 2020 S0920-9964(20)30484-9.
9. Freeman D, Taylor KM, Molodynski A, et al. Treatable clinical intervention targets for patients with schizophrenia. *Schizophr Res*. 2019; 211, 44–50.
10. McCarthy-Jones S, Thomas N, Strauss C, et al. Better than mermaids and stray dogs? Subtyping auditory verbal hallucinations and its implications for research and practice. *Schizophr Bull*, 2014; 40 Suppl 4, S275-284.
11. Stevens L, Turkington D, Drage L, et al. (2019) Investigation of a traumatic psychosis subgroup: a cluster analysis of an antipsychotic free cohort. *Psychosis*, *11*:4, 298-307,
12. England M. (2008). *Significance of Cognitive Intervention for Voice Hearers.*
13. Turkington, D, Kingdon, D, Turner, T. (2002). Effectiveness of a brief cognitive–behavioural therapy intervention in the treatment of schizophrenia. *Br J Psychiatry*, *180*(6), 523–527.
14. Waller H, Landau S , Fornells-Ambrojo M. et al (2018). Improving implementation of evidence based practice for people with psychosis through training the wider workforce: Results of the GOALS feasibility randomised controlled trial. *J Behav Ther Exp Psychiatry*, 2018 59, 121–128.
15. Hazell CM, Hayward M, Cavanagh K, et al. A systematic review and meta-analysis of low intensity CBT for psychosis. *Clin Psych Rev*, 2016*45*, 183–192.
16. Hayward M, Berry K, Bremner S, et al., Increasing access to cognitive behavioural therapy for patients with psychosis by evaluating the feasibility of a randomised trial of brief targeted cognitive behavioural therapy for distressing voices delivered by assistant psychologists. The GiVE 2 trial. 2021, *BJPsychon* Open, 4(5) E152
17. Dodgson G, Alderson-Day B, Smailes D, et al. Tailoring cognitive behavioural therapy to subtypes of voice-hearing using a novel tabletised manual: A feasibility study. *Behav Cogn Psychother*, 2020. *49*(3), 287–301.

For peer review only - http://bmjopen.bmj.com/site/about/guidelines.xhtml

18. Dodgson G, Aynsworth C, Mitrenga KJ, et al. Managing unusual sensory experiences: A feasibility trial in an At Risk Mental States for psychosis group. *Psychol Psychother*. 2021, *94*(3), 481–503.
19. Collerton D, Dudley R. A cognitive behavioural framework for the treatment of distressing visual hallucinations in older people. *Behav Cogn Psychother*, 2004,*32*(4), 443–455.
20. Haddock G, McCarron J,Tarrier N et al. Scales to measure dimensions of hallucinations and delusions: The psychotic symptom rating scales (PSYRATS). *Psychol Med*, 1999, *29*(4), 879–889.
21. Van Lieshout RJ, Goldberg JO. Quantifying self-reports of auditory verbal hallucinations in person with psychosis. Can J Beh Sci. 2007;39:73–7.
22. Dudley R, Aynsworth C, Cheetham R et al. Prevalence and characteristics of multi-modal hallucinations in people with psychosis who experience visual hallucinations. *Psychiatry Res.*, 2018, 269, 25-30.
23. Lovibond PF, Lovibond SH. The structure of negative emotional states: Comparison of the Depression Anxiety Stress Scales (DASS) with the Beck Depression and Anxiety Inventories. *Behav Res Ther*., 1995, 33(3), 335–343.
24. Neil ST, Kilbride M, Pitt L, et al. (2009). The questionnaire about the process of recovery (QPR): A measurement tool developed in collaboration with service users. *Psychosis*, 2009, *1*(2), 145–155. https://doi.org/10.1080/17522430902913450
25. Greenwood KE, Sweeney A, Williams S et al. CHoice of Outcome In Cbt for psychosEs (CHOICE): The development of a new service user-led outcome measure of CBT for psychosis. *Schizophr Bull*, 2010, 36(1), 126–135.
26. Oei TPS, Green AL. The Satisfaction With Therapy and Therapist Scale--Revised (STTS-R) for group psychotherapy: Psychometric properties and confirmatory factor analysis. *Professional Psychology: Research and Practice*, 2008,39(4), 435–442.
27. Horvath AO Greenberg LS. The development of the Working Alliance Inventory. In *The psychotherapeutic process: A research handbook* (pp. 529–556). 1986, Guilford Press.
28. Ware JE, Sherbourne CD. The MOS 36-item short-form health survey (SF-36). I. Conceptual framework and item selection. *Medical Care*,1992,*30*(6), 473–483.
29. EuroQol Research Foundation. EQ-5D-5L User Guide, 2019. Available from: https://euroqol.org/publications/user-guides. Accessed 21st January 2022
30. Flynn TN, Huynh E, Peters TJ et al. Scoring the Icecap-a capability instrument. Estimation of a UK general population tariff. *Health Econ*. 2015,24(3), 258–269.
31. Hoffmann TC, Glasziou PP, Boutron I, et al. Better reporting of interventions: Template for intervention description and replication (TIDieR) checklist and guide. *BMJ*, 2014,348, g1687.
32. Morrison AP, Pyle M, Gumley A, et al. Cognitive behavioural therapy in clozapine-resistant schizophrenia (FOCUS): An assessor-blinded, randomised controlled trial. *The Lancet Psychiatry*, 2018. *5*(8), 633–643.
33. Foster C, Startup H, Potts L, et al. A randomised controlled trial of a worry intervention for individuals with persistent persecutory delusions. *J Behav Ther Exp Psychiatry,*2010, 41(1), 45–51.
34. Moore CG, Carter RE, Nietert PJ, et al. Recommendations for planning pilot studies in clinical and translational research. *Clin Transl Sci,* 2011,*4*(5), 332–337.
35. Teare MD, Dimairo M, Shephard, N, et al. Sample size requirements to estimate key design parameters from external pilot randomised controlled trials: A simulation study. *Trials*, 2014,*15*(1), 264.

For peer review only - http://bmjopen.bmj.com/site/about/guidelines.xhtml

36. Braun V, Clarke V. *Thematic analysis: A practical guide.* 2021. SAGE Publications Ltd.
37. Patton MQ, *Qualitative Evaluation and Research Methods, Second Edition* (Second, p. 532). 1990. Sage Publications, Inc
38. Avery KNL, Williamson PR, Gamble C members of the Internal Pilot Trials Workshop supported by the Hubs for Trials Methodology Research*, et al* Informing efficient randomised controlled trials: exploration of challenges in developing progression criteria for internal pilot studies *BMJ Open* 2017;**7:**e013537
39. Bugge C, Williams B, Hagen S. A process for Decision-making after Pilot and feasibility Trials (ADePT): Development following a feasibility study of a complex intervention for pelvic organ prolapse. *Trials*, 2013, 14(1), 353.
40. O'Cathain A, Hoddinott P, Lewin S, et al. Maximising the impact of qualitative research in feasibility studies for randomised controlled trials: Guidance for researchers. *Pilot and Feasibility Studies*, 2015, *1*(1), 32.
41. Teare MD, Dimairo M, Shephard N, et al. Sample size requirements to estimate key design parameters from external pilot randomised controlled trials: A simulation study. *Trials*, 2014, *15*(1), 264.
42. Thabane L, Ma J, Chu R, al. A tutorial on pilot studies: The what, why and how. *BMC Medical Research Methodology*, 2010, 10(1), 1.

For peer review only

Figure 1 Trial flow diagram

For peer review only

For peer review only - http://bmjopen.bmj.com/site/about/guidelines.xhtml

Figure 1. Trial flow diagram

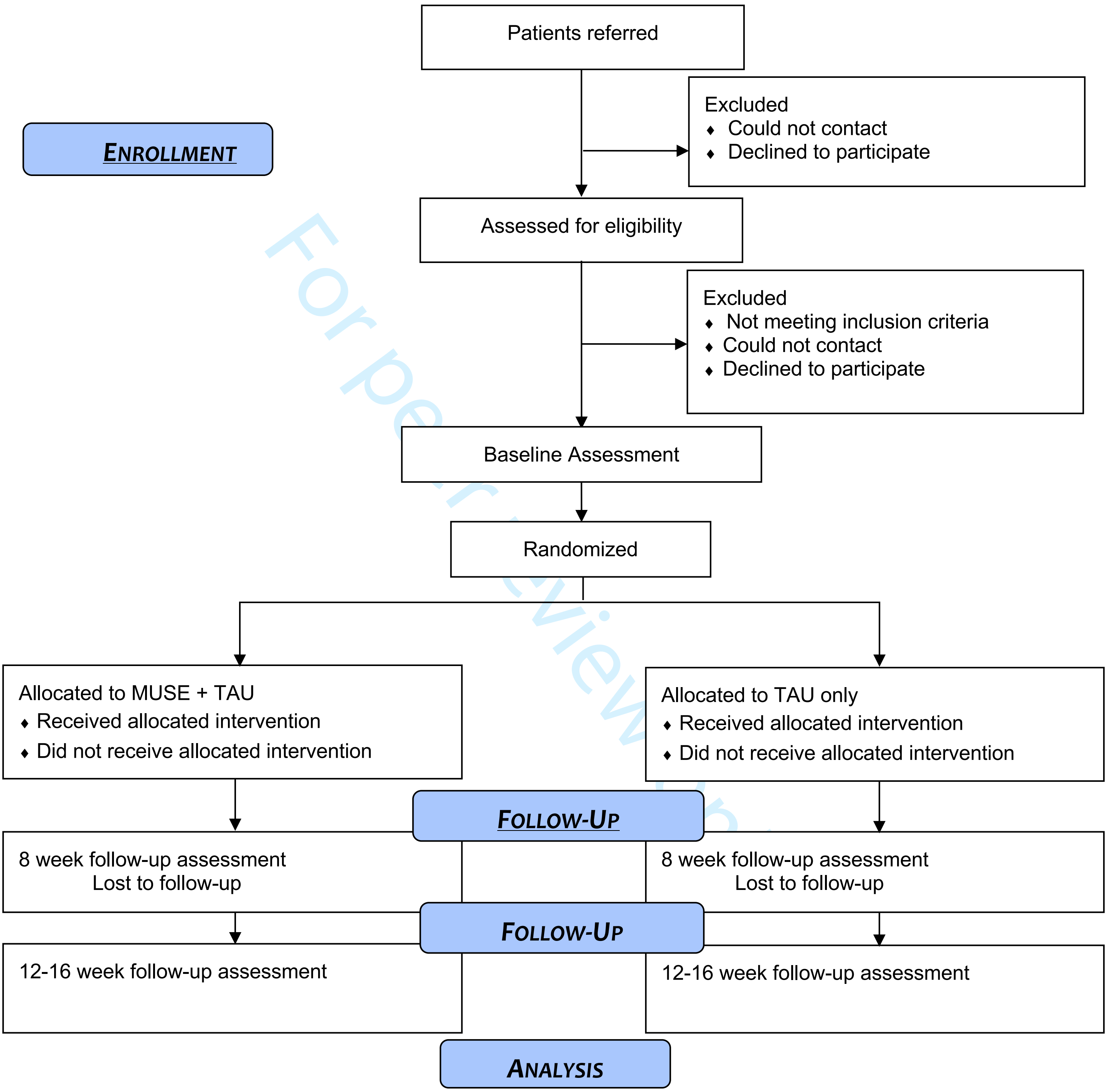

For peer review only

SPIRIT
STANDARD PROTOCOL ITEMS: RECOMMENDATIONS FOR INTERVENTIONAL TRIALS

SPIRIT 2013 Checklist: Recommended items to address in a clinical trial protocol and related documents*

Section/item	Item No	Description
**Administrative information**		
Title	1	Descriptive title identifying the study design, population, interventions, and, if applicable, trial acronym
Trial registration	2a	Trial identifier and registry name. If not yet registered, name of intended registry
Protocol version	3	Date and version identifier
Funding	4	Sources and types of financial, material, and other support
Roles and responsibilities	5a	Names, affiliations, and roles of protocol contributors
	5b	Name and contact information for the trial sponsor
	5c	Role of study sponsor and funders, if any, in study design; collection, management, analysis, and interpretation of data; writing of the report; and the decision to submit the report for publication, including whether they will have ultimate authority over any of these activities
	5d	Composition, roles, and responsibilities of the coordinating centre, steering committee, endpoint adjudication committee, data management team, and other individuals or groups overseeing the trial, if applicable (see Item 21a for data monitoring committee)
**Introduction**		
Background and rationale	6a	Description of research question and justification for undertaking the trial, including summary of relevant studies (published and unpublished) examining benefits and harms for each intervention
	6b	Explanation for choice of comparators
Objectives	7	Specific objectives
Trial design	8	Description of trial design including type of trial

For peer review only

Methods: Participants, interventions, and outcomes

Study setting	9	Description of study settings (eg, community clinic, academic hospital) and list of countries where data will be collected.
Eligibility criteria	10	Inclusion and exclusion criteria for participants. If applicable, eligibility criteria for study centres and individuals who will perform the interventions
Interventions	11a	Interventions for each group with sufficient detail to allow replication, including how and when they will be administered
	11c	Strategies to improve adherence to intervention protocols, and any procedures for monitoring adherence
	11d	Relevant concomitant care and interventions that are permitted or prohibited during the trial
Outcomes	12	Primary, secondary, and other outcomes, including the specific measurement variable, method of aggregation and time point for each outcome. Explanation of the clinical relevance of chosen efficacy and harm outcomes is strongly recommended
Participant timeline	13	Time schedule of enrolment, interventions (including any run-ins and washouts), assessments, and visits for participants. A schematic diagram is highly recommended (see Figure)
Sample size	14	Estimated number of participants needed to achieve study objectives and how it was determined, including clinical and statistical assumptions supporting any sample size calculations
Recruitment	15	Strategies for achieving adequate participant enrolment to reach target sample size

Methods: Assignment of interventions (for controlled trials)

Allocation:

Sequence generation	16a	Method of generating the allocation sequence (eg, computer-generated random numbers), and list of any factors for stratification. To reduce predictability of a random sequence, details of any planned restriction (eg, blocking) should be provided in a separate document that is unavailable to those who enrol participants or assign interventions
Allocation concealment mechanism	16b	Mechanism of implementing the allocation sequence (eg, central telephone; sequentially numbered, opaque, sealed envelopes), describing any steps to conceal the sequence until interventions are assigned

For peer review only - http://bmjopen.bmj.com/site/about/guidelines.xhtml

Implementation	16c	Who will generate the allocation sequence, who will enrol participants, and who will assign participants to interventions
Blinding (masking)	17a	Who will be blinded after assignment to interventions (eg, trial participants, care providers, outcome assessors, data analysts), and how
	17b	If blinded, circumstances under which unblinding is permissible, and procedure for revealing a participant's allocated intervention during the trial
**Methods: Data collection, management, and analysis**		
Data collection methods	18a	Plans for assessment and collection of outcome, baseline, and other trial data, including any related processes to promote data quality (eg, duplicate measurements, training of assessors) and a description of study instruments (eg, questionnaires, laboratory tests) along with their reliability and validity, if known. Reference to where data collection forms can be found, if not in the protocol
Data management		
Statistical methods	20a	Statistical methods for analysing primary and secondary outcomes.
	20b	Methods for any additional analyses
	20c	Definition of analysis population relating to protocol non-adherence (eg, as randomised analysis), and any statistical methods to handle missing data (eg, multiple imputation)
**Methods: Monitoring**		
Data monitoring	21a	Composition of data monitoring committee (DMC); summary of its role and reporting structure; statement of whether it is independent from the sponsor and competing interests; and reference to where further details about its charter can be found, if not in the protocol. Alternatively, an explanation of why a DMC is not needed
Harms	22	Plans for collecting, assessing, reporting, and managing solicited and spontaneously reported adverse events and other unintended effects of trial interventions or trial conduct
**Ethics and dissemination**		
Research ethics approval	24	Plans for seeking research ethics committee/institutional review board (REC/IRB) approval
Consent or assent	26a	Who will obtain informed consent or assent from potential trial participants or authorised surrogates, and how (see Item 32)

For peer review only - http://bmjopen.bmj.com/site/about/guidelines.xhtml

Confidentiality	27	How personal information about potential and enrolled participants will be collected, shared, and maintained in order to protect confidentiality before, during, and after the trial
Declaration of interests	28	Financial and other competing interests for principal investigators for the overall trial and each study site
Access to data	29	Statement of who will have access to the final trial dataset, and disclosure of contractual agreements that limit such access for investigators
Dissemination policy	31a	Plans for investigators and sponsor to communicate trial results to participants, healthcare professionals, the public, and other relevant groups (eg, via publication, reporting in results databases, or other data sharing arrangements), including any publication restrictions
	31c	Plans, if any, for granting public access to the full protocol, participant-level dataset, and statistical code

For peer review only

BMJ Open

Managing Unusual Sensory Experiences (MUSE) in people with first episode psychosis: A study protocol for a single blind parallel group randomised controlled feasibility trial (MUSE FEP).

Journal:	*BMJ Open*
Manuscript ID	bmjopen-2022-061827.R1
Article Type:	Protocol
Date Submitted by the Author:	14-Apr-2022

Complete List of Authors:	dudley, robert; Newcastle University; Cumbria Northumberland Tyne and Wear NHS Foundation Trust<br>Dodgson, Guy; Cumbria Northumberland Tyne and Wear NHS Foundation Trust<br>Common, Stephanie; Tees Esk and Wear Valleys NHS Foundation Trust<br>O'Grady, Lucy; Cumbria Northumberland Tyne and Wear NHS Foundation Trust<br>Watson, Florence; Tees Esk and Wear Valleys NHS Foundation Trust<br>Gibbs, Christopher; Cumbria Northumberland Tyne and Wear NHS Foundation Trust<br>Arnott, Bronia; Newcastle University Population Health Sciences Institute<br>Fernyhough, Charles; University of Durham, Department of Psychology<br>Alderson-Day, B; Durham University,<br>Ogundimu, Emmanuel; University of Durham<br>Kharatikoopaei, Ehsan; University of Durham<br>Patton, Victoria; University of Durham<br>Aynsworth, Charlotte; Cumbria Northumberland Tyne and Wear NHS Foundation Trust

**Primary Subject Heading**:	Mental health
Secondary Subject Heading:	Mental health
Keywords:	Schizophrenia & psychotic disorders < PSYCHIATRY, Adult psychiatry < PSYCHIATRY, Clinical trials < THERAPEUTICS

SCHOLARONE™
Manuscripts

Managing Unusual Sensory Experiences (MUSE) in people with first episode psychosis: A study protocol for a single blind parallel group randomised controlled feasibility trial (MUSE FEP).

Robert Dudley*[1,2], Guy Dodgson[1], Stephanie Common[3], Lucy O'Grady[1], Florence Watson[3], Christopher Gibbs[1], Bronia Arnott[2], Charles Fernyhough[4], Ben Alderson-Day[4], Emmanuel Ogundimu[4], Ehsan Kharatikoopaei [4], Victoria Patton[4], and Charlotte Aynsworth[1].

1 Cumbria, Northumberland, Tyne and Wear NHS Foundation Trust, St. Nicholas Hospital, Jubilee Road, Gosforth, Newcastle Upon Tyne, NE3 3XT, United Kingdom.

2 Population Health Sciences Institute, Faculty of Medical Sciences, Newcastle University, Baddiley Clark Building, Richardson Road, Newcastle Upon Tyne, NE1 4AX, United Kingdom.

3 Tees, Esk & Wear Valley NHS Trust, Wessex House, Falcon Court, Stockton on Tees, TS18 3TX, United Kingdom.

4. University of Durham, Stockton Road, Durham, DH1 3LE, United Kingdom.

Word count excluding title page, abstract, references and figures 3897

* Address for correspondence: Dr Robert Dudley, Gateshead Early Intervention in Psychosis service, Dryden Centre, Evistones Road, Low Fell, Gateshead, United Kingdom, NE9 5UR.

Tel 0191 223 2020 email rob.dudley@cntw.nhs.uk / rob.dudley@ncl.ac.uk

For peer review only

ABSTRACT

Introduction Hallucinations (hearing or seeing things that others do not) are a common feature of psychosis, causing significant distress and disability. Existing treatments such as Cognitive Behaviour Therapy for psychosis (CBTp) have modest benefits and there is a lack of CBTp-trained staff. Shorter, targeted treatments that focus on specific symptoms delivered by a non-specialist workforce could substantially increase access to treatment.

MUSE (Managing Unusual Sensory Experiences) explains why people have hallucinations and helps the person to develop and use coping strategies to reduce distress. MUSE focuses only on hallucinations, and treatment is short (4-6, one hour weekly sessions). It is a digital intervention, run on NHS laptops, which provides information about hallucinations in an engaging way, using audio, video, and animated content. Crucially, it is designed for use by non-specialist staff like Community Psychiatric Nurses (CPNs).

Methods and analysis The study is a two-arm feasibility Randomised Controlled Trial comparing MUSE and treatment as usual (TAU) (n=40) to TAU alone (n=40), recruiting across two NHS Trusts, using 1:1 allocation and blind assessments before and after treatment; (2 months), and at follow up (3 months). Quantitative information on recruitment rates, adherence, and completion of outcome assessments will be collected. Qualitative interviews will capture service-users' experience of therapy and clinicians' experiences of the training and supervision in MUSE. Clinicians will also be asked about factors affecting uptake, adherence, and facilitators/barriers to implementation. Analyses will focus on feasibility outcomes and provide initial estimates of intervention effects. Thematic analysis of the qualitative interviews will assess the acceptability of the training, intervention, and trial procedures.

For peer review only

Ethics and dissemination The trial has received NHS Ethical and Health Research Authority approval. Findings will be disseminated directly to participants, and services as well as through peer-reviewed publications and conference presentations.

Trial registration number ISRCTN16793301

For peer review only

For peer review only - http://bmjopen.bmj.com/site/about/guidelines.xhtml

Strengths and limitations

The MUSE FEP trial is the first digital treatment for distressing hallucinations delivered by frontline staff.

PPI involvement has been woven in throughout the development of the treatment and in the design and running of the study.

This randomised controlled multisite single-blind trial targets proposed causal factors implicated in the development of hallucinations.

The follow-up period is short, meaning that we cannot determine whether any benefits endure.

There is no active control, so we do not know what factors contribute to any change.

For peer review only

BACKGROUND

Almost all people with psychosis at some stage report hallucinations (hearing or seeing things that others do not[1]. Whilst for some these experiences can be positive[2] for many others these hallucinations can be distressing and disabling[3]. To address this need, treatments like Cognitive Behavioural Therapy for Psychosis (CBTp) are recommended[4]. A recent meta-analysis[5] indicated that CBTp has a specific and beneficial effect on hallucinations in comparison to active and inactive control conditions (Hedges *g*=0.34). Whilst welcome, the effects are still relatively modest. Moreover, access to CBTp remains limited[6] owing to the number of therapists available and the time it requires to deliver treatment[7]. CBTp is meant to be offered as 16–24 weekly sessions offered over 6-9 months.

Two approaches can help. The first is to provide briefer interventions allowing more people to receive treatment. However, the benefits of CBTp are already modest and dose of therapy is associated with outcome[8]. To retain the impact in a briefer form it is essential that shorter treatments are sufficiently potent, which will likely involve targeting key causal mechanisms[9] leading to hallucinations. The second approach is to increase access by enabling a larger and less well-trained workforce to provide treatment. The present study combines these approaches.

In terms of causal mechanisms, the exact reasons people see and hear things others do not is unclear. However, research on auditory hallucinations has shown that they take different forms that may respond to specific therapeutic approaches. A review by the International Consortium on Hallucinations Research[10] outlined at least three different potential routes to experiencing voices for people with psychosis which may be suited to different kinds of tailored intervention: *inner speech* voices (postulated to result from the misattribution of ordinary inner speech to an external agent); *hypervigilance* voices (resulting

For peer review only - http://bmjopen.bmj.com/site/about/guidelines.xhtml

from a biased attention to environmental stimuli); *memory* voices (the result of intrusions from traumatic memory[11]).

The present approach draws on this understanding and utilises it in a novel, digital toolkit called MUSE (Managing Unusual Sensory Experiences) that focuses only on hallucinations. MUSE uses psychoeducation about the currently known causal mechanisms of hallucination as means of exploring, with service-users, why their specific experiences may be happening. This knowledge is then matched to specific, tailored interventions and coping strategies that enable the person to understand and manage their experiences differently and reduce their distress. This process relies on psychoeducation as its basis, but it is more fundamentally about helping the person to change their understanding, manage their experiences better, and thus cope more effectively. MUSE is a departure from traditional CBTp as it focuses only on hallucinations and reduces the typical number of sessions to 4–6. This helps prevent the "drift" that can occur in treatment when there is not a specific target symptom or clear conceptual model underpinning treatment.

To help increase access to therapy, attempts have been made to train non-psychological therapy staff such as Community Psychiatric Nurses (CPNs) in the delivery of talking therapies[12]. However, such efforts have not demonstrated the anticipated benefits, owing to the challenge of managing high caseloads, and of providing brief training for a complex intervention and a lack of standardisation of delivery[13,14]). Recently, brief interventions (defined as less than 12 sessions[15]) provided by Assistant Psychologists for hallucinations have been trialled with some encouraging findings[16] indicating that brief targeted therapies can be provided by non-psychological therapists.

The present research continues to address this unmet need by putting high-quality treatment tools in the hands of Care Coordinators working in Early Intervention in Psychosis services (EIP). Care Coordinators (who are often Community Psychiatric Nurses (CPNs)) are

For peer review only - http://bmjopen.bmj.com/site/about/guidelines.xhtml

the most widely available workforce in EIP teams[6] potentially providing the maximum access to high-quality care. EIP care coordinators work with smaller case-loads (12-15) than in other services meaning there is capacity to deliver the treatment. Utilising the benefits of digital technology, MUSE is manualised and loaded onto an NHS laptop meaning that treatment is standardised, reducing the training required and the risk that staff will not feel confident in delivery. MUSE presents the content in a user-friendly, engaging way and is designed for joint use of therapist and service-user in the therapy session. Clinicians use prepared videos and other types of media to help explain why the person may see or hear things others do not, and to help service-users to practise coping strategies and carry out behavioural experiments to test the reality of their experiences.

An earlier version of MUSE was delivered to people with distressing voices by psychological therapists (not Care Coordinators who will deliver the toolkit in this project) in a recently completed uncontrolled single group feasibility study (MUSE PSYCHOSIS[17]). Twelve participants who completed follow-up after 10 MUSE sessions reported high treatment satisfaction and acceptability (87% & 90% mean overall ratings). The within-subject effect size for reducing auditory hallucination severity was large (d=0.7). Even greater benefits were evident on a recently completed uncontrolled study in which MUSE was used by psychological therapists with a group of people in an At Risk Mental State (ARMS) for psychosis[18]. The effect size was d=0.8 (n=19) for reduction of the intensity of auditory hallucinations, and satisfaction ratings were 90%. These studies provide encouraging evidence of the value of this approach when delivered by expert therapists.

Learning from these studies the toolkit was revised in three important ways. First, MUSE now incorporates more information about a range of hallucinations and not just auditory[19]. Second, the lived-experience input was strengthened to improve the relevance and accessibility of the MUSE materials. Third, MUSE was made easier to use for frontline

For peer review only - http://bmjopen.bmj.com/site/about/guidelines.xhtml

staff and specialist training and supervision for these staff was developed to enable them to use the MUSE toolkit.

The treatment now requires testing in a randomised trial to determine the effects on hallucinations and whether access can be improved if delivered by a non-specialist workforce. Consequently, the present study investigates the feasibility and acceptability of MUSE treatment delivered by CPNs in participants experiencing first-episode psychosis.

Objectives

The primary objective of the present study is to establish if it is possible to undertake a larger, definitive trial in the future. Therefore, we will collect data to inform the feasibility and acceptability of a targeted hallucination-focussed treatment delivered by non-psychological therapists to establish the key parameters for a future definitive trial. The study will use mixed methods to i) assess the feasibility of CPN staff training and delivery of the MUSE toolkit; ii) asses the acceptability of the MUSE toolkit to patients and staff; and to iii) collect data to inform the effectiveness of future definitive trial (including referral, recruitment, uptake and attrition rates).

METHODS AND ANALYSIS

Trial design and flow chart

The design is a prospective, parallel group, single-blind, randomised controlled feasibility trial to evaluate a novel hallucination intervention (MUSE) in addition to usual care versus usual care alone in people with psychosis attending NHS EIP services. Usual care will be recorded, but remain as standard in both groups. Assessments will be carried out at 0 (baseline), 8 weeks (post-treatment) and 12 weeks (follow-up which is 1m post-therapy but

For peer review only - http://bmjopen.bmj.com/site/about/guidelines.xhtml

with an allowance of another month if needed) by a researcher blind to treatment allocation. A number of related qualitative studies will explore the acceptability of study procedures, the delivery and experience of the intervention to participants as well as the value of the training and supervision for the staff delivering MUSE. A summary of the trial design can be seen in figure 1. The trial is registered with the ISRCTN registry: 16793301(registered 7/12/2021). There is an independent trial Data Monitoring Committee (DMC) and a Trial Steering Committee (TSC) and Patient Public Involvement group (PPI) facilitated by a co-applicant for the study with lived experience of Psychosis.

Figure one about here please

Randomisation and blinding

Sequence generation

Randomisation to the two groups will be undertaken using the web-based Sealed Envelope randomisation service (https://www.sealedenvelope.com). Randomisation will be in the ratio 1:1 to the two groups and will be stratified by site. Randomisation (at the individual level) will be independent and concealed.

Concealment mechanism

The randomization system is web-based and allocation is made known to the trial leads and trial therapists only at the point of randomisation. The allocation is dynamically generated and uses randomly varying blocks of sizes not known to the study team, so that allocation concealment is assured.

For peer review only - http://bmjopen.bmj.com/site/about/guidelines.xhtml

The researcher undertaking assessments will be blinded to group allocation, but the participants and clinician delivering the intervention cannot be blinded to condition. If there is an unblinding then future assessments will be undertaken by another assessor to maintain the blind. Breaks in blinding will be monitored and recorded.

Participants

Participants will be recruited from EIP services in two sites within the UK National Health Service (NHS) – Cumbria, Northumberland Tyne and Wear NHS Foundation Trust and Tees, Esk and Wear Valleys NHS Foundation Trust. We will recruit 80 people (aged 16 and over) with distressing hallucinations. Following referral by the clinical team, all people interested in taking part will then be approached by the research team, given information about the trial, and then screened and assessed for inclusion in the trial. Written informed consent will be obtained from each participant prior to any participation.

Inclusion criteria

Participants will:

- Be in contact with Early Intervention in Psychosis (EIP) services.
- Have an identified care coordinator.
- Meet ICD-11 criteria for schizophrenia, schizoaffective disorder or entry criteria for an EIP service.
- Have a history of auditory hallucinations for at least four weeks.
- Be aged 16 and above.
- Consider their hallucinations as a main difficulty, and would like to receive an intervention specifically for hallucinations.
- Have the capacity to provide informed consent.

For peer review only - http://bmjopen.bmj.com/site/about/guidelines.xhtml

- Be judged by their clinician to be clinically stable for the preceding 4 weeks.
- Both individuals on antipsychotic treatment, and those who decline to take medication, will be included, provided no medication changes have occurred in previous 1m (i.e., having started or stopped antipsychotic medication, or a switch to or from Clozapine).

Exclusion criteria

Participants with the following will not be eligible:

- Hallucinations/psychosis with a known biological basis.
- Insufficient command of English to complete the study procedures.
- Intellectual disability, or severe cognitive dysfunction affecting the ability to provide fully informed consent to participate.
- A primary diagnosis of substance misuse/dependency.
- Currently engaged in CBTp or received CBTp in the past 6 months.

Assessments

Independent assessors blind to therapy group will conduct all assessments at the three time points. Basic demographic and clinical data will be collected (e.g., age, gender, ethnicity, clinical diagnosis). Clinical outcomes including hallucinations, affective symptoms, social functioning, quality of life and physical health will be assessed at all three time points (baseline 0 months, post-intervention 2 months and follow-up 3-4 months)

Hallucinations are assessed using the Psychotic Symptom Rating Scales[20] which is an 11 item semi-structured interview, with 3 items being used to identify voice related distress. Also, the self-report voice-impact subscale on the Hamilton Program for Schizophrenia Voices Questionnaire (HPSVQ)[21] will also be used. Additional items asking

For peer review only

about hallucinations in non-auditory modalities (i.e., visual, somatic, olfactory and sense of presence) are assessed as well[22].

Levels of anxiety and depression (Depression, Anxiety and Stress Scales (DASS[23]) as well as perceived recovery (QPR process of recovery questionnaire[24]) are assessed. The perceived impact of the intervention (The CHoice of Outcome In Cbt for psychosEs (CHOICE[25]) is used to assess progress towards therapy-related goals. To determine therapeutic alliance & therapy acceptability, the Satisfaction with Therapy and Therapist Scale[26]) and Working Alliance inventory[27] are used. In addition, at each session, a short self-assessment form comprising items adapted from the main measure of hallucinations will monitor variations in voice frequency and distress. To help establish health economics, service use and quality of life will be measured by interview/self report (Short Form-36[28]), EQ-5D[29], perceived capability (Investigating Choice Experiments Capability Measure for Adults (ICECAP-A[30]) and from a case record review using a tool developed for the study[31].

These measures are to help identify important parameters for a future trial (i.e. completion rates and selection of best outcome measures) and to estimate the standard deviation of outcomes in order to facilitate a sample-size calculation for use within a definitive trial. Each measure has established psychometric properties for use with this population.

The MUSE FEP intervention

The intervention is a novel digital treatment for hallucinations. The treatment is divided into the following Modules:

For peer review only - http://bmjopen.bmj.com/site/about/guidelines.xhtml

1. *What are Voices?* This module provides normalising information about the frequency of voices and the factors that tend to increase voice-hearing (for example, substance misuse and sleep deprivation), along with testimonies from other voice-hearers.

2. *How the Mind Works*. This module outlines current understanding of key psychological processes such as threat detection, the importance of prediction (top-down processing) and how intrusive thoughts work.

3. *Assessment.* This module identifies the subtype of hallucination a service-user is experiencing (Inner Speech-Auditory verbal hallucination (AVH), a Memory Based AVH or a Hypervigilance AVH).

4. Inner Speech. This module provides psycho-education about the evidence that voice-hearing involves people not recognising their own inner speech. An individual understanding or formulation of voice-hearing experiences is co-produced and then targeted coping strategies and behavioural experiments are employed, such as means of interrupting and manipulating inner speech via singing or humming.

5. Memory and Trauma. This module provides psycho-education about how memories from traumatic events are more likely to be experienced as intrusive memories without contextual cues, and can therefore be experienced as belonging to the here and now. Treatment helps people manage and reframe difficult memories.

6. Hypervigilance. This module describes how our brain uses prediction to interpret the world and make sense of sensory data received. If people are expecting threatening stimuli they

For peer review only - http://bmjopen.bmj.com/site/about/guidelines.xhtml

may struggle to scrutinise poor quality sensory data and rely more heavily on predictions, whilst adopting a ‘better safe than sorry’ decision bias. Based on this understanding targeted coping strategies are employed (such as reducing arousal and stress when under threat).

7. Seeing Visions. This module draws on these other modules, and explains how our visual perceptual system can lead to mistaken perceptions, for example how easily we see faces in clouds. An individual formulation and treatment plan is then developed that normalises the experience and addresses the key cause of distress and then targeted coping strategies and behavioural experiments are employed (such as training oneself to switch attention to and from visions).

8. Sleep. This module provides psycho-education and treatment strategies about sleep, which is often a key factor in all types of unusual sensory experiences.

The intervention is delivered by a Care Coordinator on an individual basis in up to eight 1-hour sessions, though 4-6 is the usual number. There is typically one session per week. A treatment dose is defined as three or more sessions. MUSE is provided flexibly, often in people’s homes or clinics. MUSE is provided in addition to usual care. With patient consent, sessions will be audio-recorded and independently rated for quality, including fidelity and competence. MUSE is provided by care coordinators who have attended a three-day training in MUSE. They are offered regular supervision sessions. As a minimum we aim to train one team member per service that we recruit from, and they would provide the intervention in that service.

Control condition

For peer review only

The control condition is treatment-as-usual which is support from EIP services. Each participant will have a Care Coordinator, who will see the person weekly in their home usually for around 30 to 60 minutes. Where appropriate, patients are offered low dose antipsychotic medication, psychological therapies (CBTp and Family Interventions), social support, and recovery-based activities and help to manage their symptoms (but not MUSE). As per our exclusion criteria we would not accept someone currently receiving CBTp, but past the recruitment stage we will not be asking referrers to withhold any treatment. A case report form will be used to track TAU content throughout the trial based on the TIDieR checklist[31]. The MUSE intervention group will also receive TAU.

Analysis

A full statistical analysis plan will be finalised before any analysis takes place. Analyses will be based on the intention-to-treat approach. Data will be reported in line with the CONSORT - Social and Psychological Interventions (CONSORT-SPI) statement, and SPIRIT guidelines. This guidance recommends minimising the distinction between primary and secondary outcomes so all outcomes will be reported at the end of the trial.

Sample size

As the main objective is to establish feasibility parameters for a definitive trial there are no formal sample size calculations, interim analyses or stopping rules. We have estimated attrition of 12.5% based on past research of psychological therapy with people with psychosis[32] and similar brief interventions[33] meaning 70 people will complete the study. Guidance on external pilot studies indicates that samples of 35 per arm or more give a reliable estimate of the standard deviation of the outcome measure[34,35].

Statistical analysis

For peer review only - http://bmjopen.bmj.com/site/about/guidelines.xhtml

Descriptive statistics within each randomised group will be presented for baseline, end of treatment and follow up values. These will include frequency and percentages for binary and categorical variables, and means and standard deviations, or medians with interquartile range (QR), for continuous variables, along with minimum and maximum values, and frequency and percentages of missing values. There will be no tests of statistical significance or confidence intervals for differences between randomised groups on any baseline variable.

We will analyse change scores for the quantitative measures used across assessments using linear mixed effects models adjusted for site to estimate effect size between MUSE+TAU and TAU alone. The choice of mixed effect models will address the repeated measure data structure at 2 and 3 months. These will be used to inform the overall interpretation of participants' experience of therapy (combining qualitative and quantitative data). We are also interested in understanding which areas of the participant's life the intervention may impact upon, for example, distress linked to voice hearing, interpersonal functioning etc.

Qualitative analysis

Qualitative data, gathered from the structured interviews with patients and staff, will be analysed using an inductive thematic analysis[36]. This form of analysis allows for both basic and more complex themes to be classified from interview data (based on categorising sections of transcripts), based on the data themselves rather than a prior theory[37].

Health economics analysis

For a future health economic analysis we will determine the acceptability and completeness of the necessary data (care records tool, medication use, ICECAP and EQ-5D).

For peer review only - http://bmjopen.bmj.com/site/about/guidelines.xhtml

Criteria for proceeding to a full trial

We will use criteria for assessing study success and identifying feasibility factors required for delivering the definitive study [38] and follow a systematic process for decision making after pilot and feasibility trials (ADePT[39]) which helps identify criteria used to go to a full trial. These criteria are being developed with the PPI group to help determine if a full trial is warranted. We will use criteria on participant recruitment, adherence with the intervention, and retention at follow-up to assess the trial (as set out in ADePT), plus data on uptake, retention of participants, intervention fidelity and acceptability. This will use both quantitative and qualitative data derived from the study[40]. The progression criteria will be divided into three categories (green, red and amber[38]). Areas that are amenable to change before a pilot trial will be investigated and solutions discussed with the patient group for acceptability. This will help consider if a full trial is timely, necessary, and deliverable[41,42].

Adverse events

Adverse events will be reported for all participants randomised. A serious adverse event (SAE) is defined as any untoward medical occurrence that results in death, is life threatening, requires or prolongs hospitalisation or results in persistent or significant disability/incapacity. Accordingly, we will record admissions to hospital, suicide attempts, serious violent incidents and deaths. Treatment or hospitalisation for a pre-existing physical health condition, which has not worsened, does not constitute an SAE. We will record the occurrence of any SAEs reported to us and also systematically check all participants' medical records following completion of the final assessment. We will also record formal complaints regarding therapy.

For peer review only - http://bmjopen.bmj.com/site/about/guidelines.xhtml

The relevant clinical team, sponsor, and DMC will be informed of any adverse event. The DMC Chair and Sponsor will determine if it is trial related.

Patient and public involvement

Patient and Public Involvement (PPI) is facilitated by a co-applicant with personal experience of psychosis. The grant application and MUSE intervention were developed in collaboration with people who have lived with hallucinations. Following the award of the grant, a fully funded PPI group has been formed in line with recommendations of advisory groups that encourage service-user involvement in NHS research (http://www.invo.org.uk/). The PPI group will be actively involved in all stages of the research programme, for example, advising on participant recruitment, information sheets, staff training, topic guides, analysis of the qualitative interview data and dissemination of the research findings. PPI members also sit on the TSC.

Ethics and dissemination

The trial received ethical approval from the NHS Yorkshire and the Humber-Sheffield Research Ethics Committee (21/YH/0090) Health Research Authority (HRA/ HCRW) approval (IRAS 292150, The MUSE FEP trial). CNTW NHS Foundation Trust is the trial sponsor. The R&D team in the two recruitment sites (CNTW and TEWV NHS trusts) have confirmed local capacity and capability to deliver the research. The results of the trial will be published in a peer-reviewed journal and made open access. On the basis of a reasonable request the main outcome data (anonymised) will be available from the trial team after publication of the main results paper. The PPI team will help to provide feedback to all the participants, staff and services involved in the study.

For peer review only - http://bmjopen.bmj.com/site/about/guidelines.xhtml

Trial status

The trial started recruitment in June 2021 and will last for 12 months until May 2022, with final outcome data collected by September 2022. A trial paper detailing the outcomes should be submitted for publication around December 2022.

Funding statement

This paper presents independent research funded by the NIHR under its Research for Patient Benefit (RfPB) Programme (Grant Reference Number: NIHR201078). MUSE was developed with support from the Wellcome Trust (grant number WT1087201). The views expressed are those of the authors and not necessarily those of the NHS, the NIHR, or the Department of Health and Social Care. The study sponsor and funder have no role in study design; collection, management, analysis, and interpretation of data; writing of the report; and the decision to submit the report for publication.

For peer review only

Competing interests

RD receives payment for workshops in treating hallucinations and GD, SC and RD declare they are involved in running treatment studies investigating psychological therapies for psychosis. All other authors declare no competing interest. MUSE is not licensed and will be made freely available if proven to be beneficial.

Acknowledgements

We express our thanks to members of the PPI Panel for their contributions to the design of the trial and development of this protocol. We thank Adetayo Kasim and Professor Luke Vale for their input to the statistics and health economics. Kaja Mitrenga helped with set up of the

study and literature reviewing. Professor Sandra Bucci, Dr. Alison Brabban, and Dr. Valentina Short are supporting the DMC and TSC.

Contributors RD is the chief investigator, had overall responsibility for the trial design and drafted the trial protocol. CF, BAD, BA, CG, GD, SC, contributed to the study design. GD, SC, BAD and CF led the development of the treatment with substantial input from a range of clinicians and service users. CG is responsible for Patient and Public Involvement, with support from VP. EK and EO are responsible for the statistical analysis. CA, FW and LOG are co-ordinating the trial and are responsible for recruitment. All authors read and approved the final trial protocol.

For peer review only

References

1. McCarthy-Jones S, Smailes D, Corvin A, et al. Occurrence and co-occurrence of hallucinations by modality in schizophrenia spectrum disorders. *Psychiatry Res.*, 2017; 252, 154-60.
2. Beavan V, Read J, Cartwright C. The prevalence of voice-hearers in the general population: a literature review. *J Ment Health.* 2011;20:281–92.
3. Morrison AP, Nothard S, Bowe SE et al. Interpretations of voices in patients with hallucinations and non-patient controls: a comparison and predictors of distress inpatients. *Behav Res Ther*. 2004;42:1315–23.
4. National Institute for Clinical Excellence. Psychosis and schizophrenia in adults: prevention and management (CG178). NICE, 2014.
5. Turner DT, Burger S, Smit F, et al. What Constitutes Sufficient Evidence for Case Formulation-Driven CBT for Psychosis? Cumulative Meta-analysis of the Effect on Hallucinations and Delusions. *Schizophr Bull.* 2020 Mar 27;46(5):1072–85.
6. The Royal College of Psychiatrists. National Clinical Audit of Psychosis – National Report for the Core Audit. London: Healthcare Quality Improvement Partnership; 2018. https://www.hqip.org.uk/wp-content/ uploads/2018/07/NCAP-National-report-for-core-audit-2018-FINAL.pdf. .
7. Ince P, Haddock G, Tai S. A systematic review of the implementation of recommended psychological interventions for schizophrenia: rates, barriers, and improvement strategies. *Psychol Psychother.* 2016;89:324–50.
8. Spencer H, Dudley R, Freeston M, et al. What are the essential ingredients of a CBT case conceptualization for voices and delusions in schizophrenia spectrum disorders? A study of expert consensus. *Schizophr Res*. 2020 S0920-9964(20)30484-9.
9. Freeman D, Taylor KM, Molodynski A, et al. Treatable clinical intervention targets for patients with schizophrenia. *Schizophr Res*. 2019; 211, 44–50.
10. McCarthy-Jones S, Thomas N, Strauss C, et al. Better than mermaids and stray dogs? Subtyping auditory verbal hallucinations and its implications for research and practice. *Schizophr Bull*, 2014; 40 Suppl 4, S275-284.
11. Stevens L, Turkington D, Drage L, et al. (2019) Investigation of a traumatic psychosis subgroup: a cluster analysis of an antipsychotic free cohort. *Psychosis*, *11*:4, 298-307,
12. England M. (2008). *Significance of Cognitive Intervention for Voice Hearers.*
13. Turkington, D, Kingdon, D, Turner, T. (2002). Effectiveness of a brief cognitive–behavioural therapy intervention in the treatment of schizophrenia. *Br J Psychiatry*, *180*(6), 523–527.
14. Waller H, Landau S , Fornells-Ambrojo M. et al (2018). Improving implementation of evidence based practice for people with psychosis through training the wider workforce: Results of the GOALS feasibility randomised controlled trial. *J Behav Ther Exp Psychiatry*, 2018 59, 121–128.
15. Hazell CM, Hayward M, Cavanagh K, et al. A systematic review and meta-analysis of low intensity CBT for psychosis. *Clin Psych Rev*, 2016*45*, 183–192.
16. Hayward M, Berry K, Bremner S, et al., Increasing access to cognitive behavioural therapy for patients with psychosis by evaluating the feasibility of a randomised trial of brief targeted cognitive behavioural therapy for distressing voices delivered by assistant psychologists. The GiVE 2 trial. 2021, *BJPsychon* Open, 4(5) E152
17. Dodgson G, Alderson-Day B, Smailes D, et al. Tailoring cognitive behavioural therapy to subtypes of voice-hearing using a novel tabletised manual: A feasibility study. *Behav Cogn Psychother*, 2020. *49*(3), 287–301.

For peer review only

18. Dodgson G, Aynsworth C, Mitrenga KJ, et al. Managing unusual sensory experiences: A feasibility trial in an At Risk Mental States for psychosis group. *Psychol Psychother.* 2021, *94*(3), 481–503.
19. Collerton D, Dudley R. A cognitive behavioural framework for the treatment of distressing visual hallucinations in older people. *Behav Cogn Psychother*, 2004,*32*(4), 443–455.
20. Haddock G, McCarron J,Tarrier N et al. Scales to measure dimensions of hallucinations and delusions: The psychotic symptom rating scales (PSYRATS). *Psychol Med*, 1999, *29*(4), 879–889.
21. Van Lieshout RJ, Goldberg JO. Quantifying self-reports of auditory verbal hallucinations in person with psychosis. Can J Beh Sci. 2007;39:73–7.
22. Dudley R, Aynsworth C, Cheetham R et al. Prevalence and characteristics of multi-modal hallucinations in people with psychosis who experience visual hallucinations. *Psychiatry Res.*, 2018, 269, 25-30.
23. Lovibond PF, Lovibond SH. The structure of negative emotional states: Comparison of the Depression Anxiety Stress Scales (DASS) with the Beck Depression and Anxiety Inventories. *Behav Res Ther.*, 1995, 33(3), 335–343.
24. Neil ST, Kilbride M, Pitt L, et al. (2009). The questionnaire about the process of recovery (QPR): A measurement tool developed in collaboration with service users. *Psychosis*, 2009, *1*(2), 145–155. https://doi.org/10.1080/17522430902913450
25. Greenwood KE, Sweeney A, Williams S et al. CHoice of Outcome In Cbt for psychosEs (CHOICE): The development of a new service user-led outcome measure of CBT for psychosis. *Schizophr Bull*, 2010, 36(1), 126–135.
26. Oei TPS, Green AL. The Satisfaction With Therapy and Therapist Scale--Revised (STTS-R) for group psychotherapy: Psychometric properties and confirmatory factor analysis. *Professional Psychology: Research and Practice*, 2008,39(4), 435–442.
27. Horvath AO Greenberg LS. The development of the Working Alliance Inventory. In *The psychotherapeutic process: A research handbook* (pp. 529–556). 1986, Guilford Press.
28. Ware JE, Sherbourne CD. The MOS 36-item short-form health survey (SF-36). I. Conceptual framework and item selection. *Medical Care*,1992,*30*(6), 473–483.
29. EuroQol Research Foundation. EQ-5D-5L User Guide, 2019. Available from: https://euroqol.org/publications/user-guides. Accessed 21st January 2022
30. Flynn TN, Huynh E, Peters TJ et al. Scoring the Icecap-a capability instrument. Estimation of a UK general population tariff. *Health Econ.* 2015,24(3), 258–269.
31. Hoffmann TC, Glasziou PP, Boutron I, et al. Better reporting of interventions: Template for intervention description and replication (TIDieR) checklist and guide. *BMJ*, 2014,348, g1687.
32. Morrison AP, Pyle M, Gumley A, et al. Cognitive behavioural therapy in clozapine-resistant schizophrenia (FOCUS): An assessor-blinded, randomised controlled trial. *The Lancet Psychiatry*, 2018. *5*(8), 633–643.
33. Foster C, Startup H, Potts L, et al. A randomised controlled trial of a worry intervention for individuals with persistent persecutory delusions. *J Behav Ther Exp Psychiatry,*2010, 41(1), 45–51.
34. Moore CG, Carter RE, Nietert PJ, et al. Recommendations for planning pilot studies in clinical and translational research. *Clin Transl Sci,* 2011,*4*(5), 332–337.
35. Teare MD, Dimairo M, Shephard, N, et al. Sample size requirements to estimate key design parameters from external pilot randomised controlled trials: A simulation study. *Trials*, 2014,*15*(1), 264.

For peer review only - http://bmjopen.bmj.com/site/about/guidelines.xhtml

36. Braun V, Clarke V. *Thematic analysis: A practical guide.* 2021. SAGE Publications Ltd.
37. Patton MQ, *Qualitative Evaluation and Research Methods, Second Edition* (Second, p. 532). 1990. Sage Publications, Inc
38. Avery KNL, Williamson PR, Gamble C members of the Internal Pilot Trials Workshop supported by the Hubs for Trials Methodology Research*, et al* Informing efficient randomised controlled trials: exploration of challenges in developing progression criteria for internal pilot studies *BMJ Open* 2017;**7:**e013537
39. Bugge C, Williams B, Hagen S. A process for Decision-making after Pilot and feasibility Trials (ADePT): Development following a feasibility study of a complex intervention for pelvic organ prolapse. *Trials*, 2013, 14(1), 353.
40. O'Cathain A, Hoddinott P, Lewin S, et al. Maximising the impact of qualitative research in feasibility studies for randomised controlled trials: Guidance for researchers. *Pilot and Feasibility Studies*, 2015, *1*(1), 32.
41. Teare MD, Dimairo M, Shephard N, et al. Sample size requirements to estimate key design parameters from external pilot randomised controlled trials: A simulation study. *Trials*, 2014, *15*(1), 264.
42. Thabane L, Ma J, Chu R, al. A tutorial on pilot studies: The what, why and how. *BMC Medical Research Methodology*, 2010, 10(1), 1.

For peer review only

Figure 1 Trial flow diagram

For peer review only

For peer review only - http://bmjopen.bmj.com/site/about/guidelines.xhtml

Figure 1. Trial flow diagram

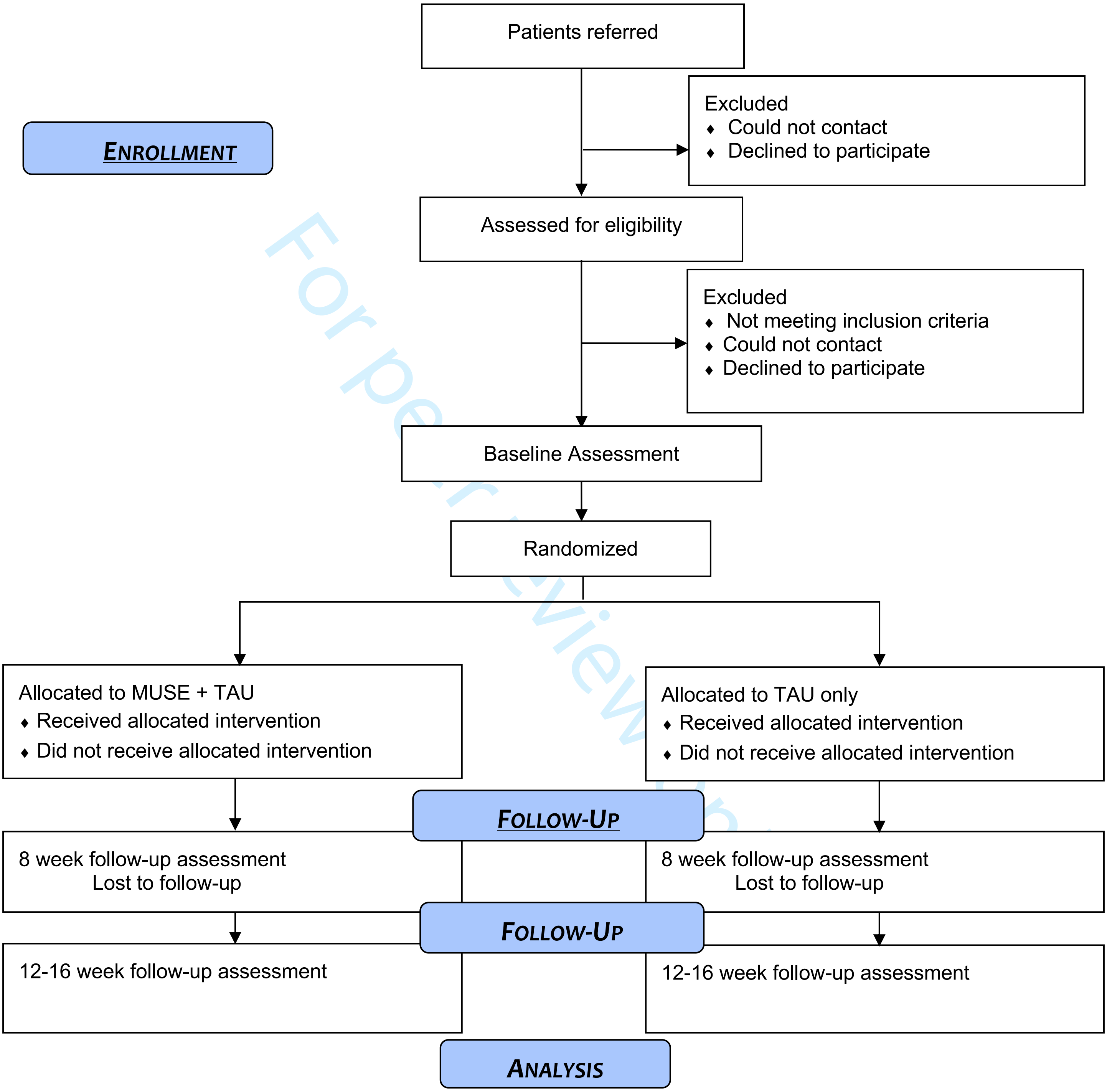

For peer review only

SPIRIT

STANDARD PROTOCOL ITEMS: RECOMMENDATIONS FOR INTERVENTIONAL TRIALS

SPIRIT 2013 Checklist: applied to MUSE FEP study

Section/item	Item No	Description
**Administrative information**		
Title	1	Descriptive title identifying the study design, population, interventions, and, if applicable, trial acronym
Trial registration	2a	Trial identifier and registry name.
Protocol version	3	Date and version identifier
Funding	4	Sources and types of financial, material, and other support
Roles and responsibilities	5a	Names, affiliations, and roles of protocol contributors
	5b	Name and contact information for the trial sponsor
	5c	Role of study sponsor and funders, if any, in study design; collection, management, analysis, and interpretation of data; writing of the report; and the decision to submit the report for publication, including whether they will have ultimate authority over any of these activities
	5d	Composition, roles, and responsibilities of the coordinating centre, steering committee, endpoint adjudication committee, data management team, and other individuals or groups overseeing the trial, if applicable (see Item 21a for data monitoring committee)
**Introduction**		
Background and rationale	6a	Description of research question and justification for undertaking the trial, including summary of relevant studies (published and unpublished) examining benefits and harms for each intervention
	6b	Explanation for choice of comparators
Objectives	7	Specific objectives
Trial design	8	Description of trial design including type of trial

Methods: Participants, interventions, and outcomes

For peer review only

Study setting	9	Description of study settings (eg, community clinic, academic hospital) and list of countries where data will be collected.
Eligibility criteria	10	Inclusion and exclusion criteria for participants. If applicable, eligibility criteria for study centres and individuals who will perform the interventions
Interventions	11a	Interventions for each group with sufficient detail to allow replication, including how and when they will be administered
	11c	Strategies to improve adherence to intervention protocols, and any procedures for monitoring adherence
	11d	Relevant concomitant care and interventions that are permitted or prohibited during the trial
Outcomes	12	Primary, secondary, and other outcomes, including the specific measurement variable, method of aggregation and time point for each outcome. Explanation of the clinical relevance of chosen efficacy and harm outcomes is strongly recommended
Participant timeline	13	Time schedule of enrolment, interventions (including any run-ins and washouts), assessments, and visits for participants. A schematic diagram is highly recommended (see Figure)
Sample size	14	Estimated number of participants needed to achieve study objectives and how it was determined, including clinical and statistical assumptions supporting any sample size calculations
Recruitment	15	Strategies for achieving adequate participant enrolment to reach target sample size

Methods: Assignment of interventions (for controlled trials)

Allocation:

Sequence generation	16a	Method of generating the allocation sequence (eg, computer-generated random numbers), and list of any factors for stratification. To reduce predictability of a random sequence, details of any planned restriction (eg, blocking) should be provided in a separate document that is unavailable to those who enrol participants or assign interventions
Allocation concealment mechanism	16b	Mechanism of implementing the allocation sequence (eg, central telephone; sequentially numbered, opaque, sealed envelopes), describing any steps to conceal the sequence until interventions are assigned
Implementation	16c	Who will generate the allocation sequence, who will enrol participants, and who will assign participants to interventions

Blinding (masking)	17a	Who will be blinded after assignment to interventions (eg, trial participants, care providers, outcome assessors, data analysts), and how
	17b	If blinded, circumstances under which unblinding is permissible, and procedure for revealing a participant's allocated intervention during the trial

Methods: Data collection, management, and analysis

Data collection methods	18a	Plans for assessment and collection of outcome, baseline, and other trial data, including any related processes to promote data quality (eg, duplicate measurements, training of assessors) and a description of study instruments (eg, questionnaires, laboratory tests) along with their reliability and validity, if known. Reference to where data collection forms can be found, if not in the protocol
Data management		
Statistical methods	20a	Statistical methods for analysing primary and secondary outcomes.
	20b	Methods for any additional analyses
	20c	Definition of analysis population relating to protocol non-adherence (eg, as randomised analysis), and any statistical methods to handle missing data (eg, multiple imputation)

Methods: Monitoring

Data monitoring	21a	Composition of data monitoring committee (DMC); summary of its role and reporting structure; statement of whether it is independent from the sponsor and competing interests; and reference to where further details about its charter can be found, if not in the protocol. Alternatively, an explanation of why a DMC is not needed
Harms	22	Plans for collecting, assessing, reporting, and managing solicited and spontaneously reported adverse events and other unintended effects of trial interventions or trial conduct

Ethics and dissemination

Research ethics approval	24	Plans for seeking research ethics committee/institutional review board (REC/IRB) approval
Consent or assent	26a	Who will obtain informed consent or assent from potential trial participants or authorised surrogates, and how (see Item 32)

For peer review only - http://bmjopen.bmj.com/site/about/guidelines.xhtml

Confidentiality	27	How personal information about potential and enrolled participants will be collected, shared, and maintained in order to protect confidentiality before, during, and after the trial
Declaration of interests	28	Financial and other competing interests for principal investigators for the overall trial and each study site
Access to data	29	Statement of who will have access to the final trial dataset, and disclosure of contractual agreements that limit such access for investigators
Dissemination policy	31a	Plans for investigators and sponsor to communicate trial results to participants, healthcare professionals, the public, and other relevant groups (eg, via publication, reporting in results databases, or other data sharing arrangements), including any publication restrictions
	31c	Plans, if any, for granting public access to the full protocol, participant-level dataset, and statistical code

For peer review only
