# Supplementary material for: Managing Unusual Sensory Experiences in People with First-Episode Psychosis (MUSE FEP): a study protocol for a single-blind parallel-group randomised controlled feasibility trial
Source: BMJ Open. 2022 May 16;12(5):e061827. doi: 10.1136/bmjopen-2022-061827 (PMC9114953; doi:10.1136/bmjopen-2022-061827)
Supplement: Reviewer comments [file bmjopen-2022-061827.reviewer_comments.pdf]

PEER REVIEW HISTORY

BMJ Open publishes all reviews undertaken for accepted manuscripts. Reviewers are asked to complete a checklist review form (**http://bmjopen.bmj.com/site/about/resources/checklist.pdf**) and are provided with free text boxes to elaborate on their assessment. These free text comments are reproduced below.

ARTICLE DETAILS

**TITLE (PROVISIONAL)**	Managing Unusual Sensory Experiences (MUSE) in people with first episode psychosis: A study protocol for a single blind parallel group randomised controlled feasibility trial (MUSE FEP).
**AUTHORS**	Dudley, Robert; Dodgson, Guy; Common, Stephanie; O'Grady, Lucy; Watson, Florence; Gibbs, Christopher; Arnott, Bronia; Fernyhough, Charles; Alderson-Day, B; Ogundimu, Emmanuel; Kharatikoopaei, Ehsan; Patton, Victoria; Aynsworth, Charlotte

This article was not externally reviewed at BMJ Open. Protocols that have been independently assessed prior to submission to BMJ Open are usually fast-tracked to publication on the grounds that further substantial changes will not be possible. This independent assessment will usually be external, independent review for both a substantial grant award from a non-commercial or government funder and ethics approval.
